# Reliability measures in knowledge structure theory

**DOI:** 10.1111/bmsp.70013

**Published:** 2025-11-01

**Authors:** Debora de Chiusole, Andrea Spoto, Umberto Granziol, Luca Stefanutti

**Affiliations:** ^1^ FISPPA Department University of Padua Padua Italy; ^2^ Department of General Psychology University of Padua Padua Italy; ^3^ Centro di Ateneo Servizi Clinici Universitari Psicologici (SCUP) University of Padua Padua Italy; ^4^ IRCCS San Camillo Hospital Venice Italy

**Keywords:** assessment, basic local independence model, classification, information theory, knowledge structures, latent class analysis, reliability

## Abstract

In knowledge structure theory (KST) framework, this study evaluates the reliability of knowledge state estimation by introducing two key measures: the expected accuracy rate and the expected discrepancy. The accuracy rate quantifies the likelihood that the estimated knowledge state aligns with the true state, while the expected discrepancy measures the average deviation when misclassification occurs. To support the theoretical framework, we provide an in‐depth discussion of these indices, supplemented by two simulation studies and an empirical example. The simulation results reveal a trade‐off between the number of items and the size of the knowledge structure. Specifically, smaller structures exhibit consistent accuracy across different error levels, while larger structures show increasing discrepancies as error rates rise. Nevertheless, accuracy improves with a greater number of items in larger structures, mitigating the impact of errors. Additionally, the expected discrepancy analysis shows that when misclassification occurs, the estimated state is generally close to the true one, minimizing the effect of errors in the assessment. Finally, an empirical application using real assessment data demonstrates the practical relevance of the proposed measures. This suggests that KST‐based assessments provide reliable and meaningful diagnostic information, highlighting their potential for use in educational and psychological testing.

## 1 INTRODUCTION

The assessment of individual's ability is a topic that has been widely studied in psychology in the last century contributing in the development and formalization of a number of different approaches. Among them, Classical Test Theory (CTT; Gulliksen, 2013; Novick, 1965) and the Rasch model (Rasch, 1960) represent the most widespread methods. Both of them use the observed sum score as a direct indicator of ability. Nonetheless, more recently, some further approaches based on a non‐numerical measure of individual's ability such as Knowledge Structure Theory (KST; Doignon & Falmagne, 1985; Falmagne & Doignon, 2010; Heller & Stefanutti, 2024) and Cognitive Diagnostic Models (CDM; Tatsuoka, 1985; Templin & Henson, 2006) have been proposed and deeply studied. In particular, KST provides a multidimensional, non‐numerical representation of knowledge, where the so‐called knowledge state is the set of all the problems an individual masters within a specific domain.

While the numerical and non‐numerical approaches to the measure of ability, evidently, differ on several conceptual, procedural and application aspects, some common instances and challenges exist. Among them, in this research we focus on one of the most important requirements that any assessment, either numerical or non‐numerical, must present, that is, reliability. The definition of reliability differs between the two approaches, depending on the specific assumptions that characterize each of them. For instance, in CTT, the reliability of a test depends on how noisy the observed scores of individuals are, where noise corresponds to the variance of an error term. On the other hand, in KST the reliability of the assessment is measured with respect to the estimated knowledge states.

In this respect, although KST is a well‐established measurement approach, for a long time it lacked an overall reliability index. In the past, reliability in KST has been assessed indirectly, through error parameter estimation for individual items or, for adaptive assessment methods, by using the so‐called extra question procedure (Falmagne et al., 2013). Recently, de Chiusole et al. (2024) began to address this significant gap. They first explored the feasibility of applying existing CTT methods to estimate reliability within the KST framework. However, they showed that the fundamental assumptions of CTT are incompatible with all those theoretical approaches, like KST, where the error rate is not independent of the ‘true score’. For this reason, traditional reliability indices resulted unsuitable for KST. Drawing upon information theory (Shannon, 1948), de Chiusole et al. (2024) introduced two novel reliability indices specifically designed for KST: the response pattern reliability (RP‐Reliability) and the knowledge state reliability (KS‐Reliability). RP‐Reliability provides a measure of the reliability of the observed response patterns, while KS‐Reliability provides a measure of the reliability of the estimated knowledge state.

Continuing the work by de Chiusole et al. (2024), the present research analytically derives and studies the probability distribution of the discrepancy between the true and estimated knowledge states. This distribution gives rise to a series of interesting reliability indices. Among them, an *expected accuracy rate* is obtained as the probability that, in an individual assessment, the estimated knowledge state matches the true one.

The paper is structured as follows. The next section introduces some background on basic KST and on the already existing methods for reliability estimation in this framework. Section 3 introduces the new indices for the estimation of reliability within the KST framework. Sections 4 and 5 study the asymptotic behaviour of the new indices and test the indices on simulated finite samples. An empirical example of the application of the indices is provided in Section 6. Finally, some comments, remarks and future directions are discussed in Section 7.

## 2 BACKGROUND: BASIC KST AND STATE OF THE ART OF THE STUDY OF RELIABILITY

Knowledge Space Theory (KST; Doignon & Falmagne, 1985, 1999) is a theoretical framework developed to represent and assess human knowledge in a formal, structured and non‐numerical way. At its core, KST assumes that the knowledge of individuals can be described in terms of the specific problems or items they are capable of solving within a domain of interest. This domain is formalized as a set Q of items (e.g. exercises, test questions or diagnostic criteria). In the present article, we assume Q to be finite.

Within this framework, a *knowledge state* is the subset K⊆Q that contains exactly the items q∈Q an individual masters. For example, if the domain is Q={a,b,c}, a knowledge state could be K={a,b}, indicating that the individual can solve items a and b, but not c. Crucially, not all subsets of Q necessarily represent feasible or coherent knowledge states. Only a selected collection of them—those that are theoretically or empirically justifiable—form what is called a *knowledge structure*, denoted $𝒦\subseteq 2^{Q}$. A knowledge structure must at least include the empty set ∅ (representing individuals who master no items) and Q (representing complete mastery).

To illustrate this concept more concretely, suppose that the knowledge structure 𝒦 over Q={a,b,c} is defined as:

𝒦={∅,{a},{a,b},{a,c},{a,b,c}}.

This means that only these five subsets of Q are knowledge states. In contrast, subsets such as {b} or {b,c} are *not* knowledge states.

In the first phase of the development of KST, the deterministic components introduced above were formalized, while the probabilistic parts of the theory (i.e. parameters estimation and adaptive assessments) were deeply studied in the following years (Falmagne & Doignon, 1988a, 1988b). This second phase led to the definition of some probabilistic models, among which the restricted latent class model called the basic local independence model (BLIM; Falmagne & Doignon, 1988a) is by far the most widely adopted in practical applications. Given that the knowledge state K of an individual is latent, what can be observed when an assessment of knowledge is carried out is the *response pattern* $R\subseteq 2^{Q}$ of each evaluated individual. According to the BLIM the probability P(R) of a given response pattern R⊆Q is:

(1) $$P(R)=\underset{K\in 𝒦}{\sum }P(R|K)\pi_{K}.$$

In Equation (1), P(R|K) is the conditional probability of the response pattern given a state K∈𝒦, while $\pi_{K}$ is the probability of a state K. Notice that in the BLIM the answers to the items are locally independent given the knowledge state of the person. The conditional probability P(R|K) is determined by two error probability parameters of each item q∈Q, namely, the *careless error* $\beta_{q}$ and the *lucky guess* $\eta_{q}$, in the interval [0,1), with $\eta_{q}+\beta_{q}<1$. The conditional probability of the pattern R given the state K is determined by the error parameters according to the following Equation (2):

(2) $$P(R|K)=\left(\underset{q\in K\setminus R}{\prod }\beta_{q}\right)\left(\underset{q\in K\cap R}{\prod }(1-\beta_{q})\right)\left(\underset{q\in R\setminus K}{\prod }\eta_{q}\right)\left(\underset{q\in Q\setminus (K\cup R)}{\prod }(1-\eta_{q})\right).$$

Continuing the example introduced above, let us suppose that the response pattern of an individual is R={a,c} and that their knowledge state is K={b,c}. Then, by applying Equation (2), $P(R|K)=\eta_{a}\beta_{b}(1-\beta_{c})$, because a∈R∖K (a lucky guess), b∈K∖R (a careless error) and c∈K∩R (an error not due to carelessness).

For many years the study of reliability in KST lacked a global index and, to indirectly estimate the reliability of each single item, $\eta_{q}$ and $\beta_{q}$ values were used, with the rule of thumb interpreting low values of these parameters as an indicator of good reliability. Given the particular nature of KST, reliability can be evaluated also with respect to the assessment conducted to estimate the knowledge state of an individual. Within this specific framework, all the main methods available to estimate assessment reliability refer to the *extra question procedure* (Falmagne et al., 2013). In this method, a question not related to the ongoing assessment is administered and the observed answer is used in different ways to estimate the reliability of the overall procedure (Falmagne et al., 2013).

As mentioned above, de Chiusole et al. (2024) proposed two reliability indices for KST: the *RP‐Reliability* provides a measure of the reliability of the observed response patterns; the *KS‐Reliability* provides a measure of the reliability of a probabilistic knowledge structure as a means to assess individual knowledge states. Both indices are defined within an information theoretic framework. Within this framework the RP‐Reliability is conceptualized as the amount of information the knowledge state provides for completely recovering the observed response pattern. Formally:

(3) $$U(K|R)=1-\frac{H(R|K)}{H(R)},$$

where R and K are random variables with realizations in $2^{Q}$ and 𝒦, respectively, H(R) is the Shannon's entropy of the random variable R, and H(R|K) is the conditional entropy of R given K. The value of U(K|R) varies in the interval [0,1]. In particular, it is 0 when R and K are two independent random variables, and it is 1 when there is no random error.

On the other hand, the KS‐Reliability is conceived as the amount of information needed for describing the state K which is accounted for by the observed response pattern R. In this sense, KS‐Reliability can be regarded as an indicator of the reliability of real assessment and it is formally defined as follows:

(4) $$U(R|K)=\frac{H(R)-H(R|K)}{H(K)}.$$

Like U(K|R), the index U(R|K) also ranges in the interval [0,1]. The index is minimum when H(R|K)=H(R), that is when the random variables R and K are independent, while it is maximum when H(R|K)=0. In de Chiusole et al. (2024) it is shown that this happens if and only if all the item error parameters (i.e. $\eta_{q}$ and $\beta_{q}$) are zero, so that there is an identity between the observed response pattern and the latent knowledge state.

## 3 THE NEW KST‐BASED RELIABILITY INDICES

In the assessment of knowledge structures, a fundamental question concerns the reliability of the estimated knowledge state. The accuracy of such estimations can be evaluated by comparing the estimated state with the true, albeit unknown, knowledge state of the individual. However, beyond the mere correctness of the estimation, it is also important to quantify the extent of potential discrepancies when errors occur.

In the following sections, we introduce two key measures to address these aspects. First, we define the expected accuracy rate, which represents the probability that the estimated knowledge state coincides with the true one.

Second, we consider the expected discrepancy between the estimated and true knowledge states. Even when accuracy is not perfect, a small discrepancy between the two states may still indicate a useful approximation. To quantify this, we introduce a measure based on the symmetric difference between knowledge states, allowing us to assess how far, on average, the estimated state deviates from the true one.

Together, these measures offer a comprehensive perspective on the reliability of knowledge state estimation, balancing both correctness and the severity of misclassifications.

### 3.1 The expected accuracy rate

Suppose that knowledge assessment is performed with the knowledge structure (Q,𝒦). In this section we shall consider situations where all items in Q are administered to an individual whose true, though unknown knowledge state is K∈𝒦. A response pattern R is observed, and the so‐called *modal state* $\hat{K}$ is computed as an estimate of the true state K. The modal state is obtained as the mode of the posterior likelihood distribution P(K|R) such that, for any K∈𝒦 and any $R\in 2^{Q}$,

(5) $$P(K|R)=\frac{P(R|K)\pi_{K}}{\sum_{L\in 𝒦}P(R|L)\pi_{L}}.$$

We define the *accuracy rate* as the probability that the modal state $\hat{K}$ equals the true state K. Let K be a random variable with realizations in 𝒦, representing the true latent knowledge state of an individual randomly sampled from the reference population. The probability of sampling an individual in knowledge state K∈𝒦 is $P(K=K)=\pi_{K}$. Let moreover $\hat{K}$ be a second random variable with realizations in 𝒦, representing the outcome of the assessment. The probability that $\hat{K}$ is the outcome of the assessment is denoted $P(\hat{K}=\hat{K})$.

An assessment is classified as ‘correct’ if the outcome $\hat{K}$ equals the true knowledge state K of an individual. We want to compute the probability $P(\hat{K}=K)$ of a correct assessment, that is the accuracy rate. The joint probability that both K and $\hat{K}$ equal a specific knowledge state K∈𝒦 is

$$P(\hat{K}=K,K=K)=P(\hat{K}=K|K=K)\pi_{K},$$

where $P(\hat{K}=K|K=K)$ is the conditional probability that the outcome of the assessment is K, given that the true state is K. This last can be written as

$$\begin{array}{ll} P(\hat{K}=K|K=K) & =\underset{R\in 2^{Q}}{\sum }P(\hat{K}=K|R,K)P(R|K)\;\\ & =\underset{R\in 2^{Q}}{\sum }P(\hat{K}=K|R)P(R|K),\; \end{array}$$

where $P(\hat{K}=K|R)$ is the conditional probability that the outcome of the assessment is K, given that the observed response pattern is R, and P(R|K) is the conditional probability of response pattern R, given that the true state is K. Notice that $P(\hat{K}=K|R,K)=P(\hat{K}=K|R)$, because the estimated and the true knowledge states are conditionally independent, given the observed response pattern. This last conditional probability is a function of the $\beta_{q}$ and $\eta_{q}$ error probabilities of the items. As for the probability $P(\hat{K}=K|R)$, we have^1^

$$P(\hat{K}=K|R)=\left\{\begin{array}{cc} 1/|{𝒦}_{R}|\; & \text{if}\;R\in {ℛ}_{K}\\ 0\; & \text{if}\;R\in 2^{Q}\setminus {ℛ}_{K}, \end{array}\right.$$

where, for every K∈𝒦

$${ℛ}_{K}=\left\{R\in 2^{Q}:P(K|R)=\underset{L\in 𝒦}{\max}P(L|R)\right\},$$

is the set of all those response patterns that have K as the modal state, and for every $R\in 2^{Q}$,

$${𝒦}_{R}=\left\{K\in 𝒦:P(K|R)=\underset{L\in 𝒦}{\max}P(L|R)\right\},$$

is the set of all the knowledge states that are modal for a given response pattern. In other words, it is assumed that, if the modal state is not unique then one of the alternatives is selected with equal probability.

Then, we obtain

$$P(\hat{K}=K|K=K)=\underset{R\in {ℛ}_{K}}{\sum }\frac{P(R|K)}{\left|{𝒦}_{R}\right|},$$

and therefore, the joint probability is

$$P(\hat{K}=K,K=K)=\underset{R\in {ℛ}_{K}}{\sum }\frac{P(R|K)\pi_{K}}{\left|{𝒦}_{R}\right|}.$$

The probability $P(\hat{K}=K)$ we are looking for is then

(6) $$\begin{array}{ll} P(\hat{K}=K) & =\underset{K\in 𝒦}{\sum }P(\hat{K}=K,K=K)\\ & =\underset{K\in 𝒦}{\sum }\underset{R\in {ℛ}_{K}}{\sum }\frac{P(R|K)\pi_{K}}{\left|{𝒦}_{R}\right|}.\; \end{array}$$

We refer to the probability $\rho =P(\hat{K}=K)$ as the *accuracy rate*.

It should be observed that the accuracy rate is obtained as a weighted sum of conditional probabilities $P(\hat{K}=K|K=K)$ across all knowledge states K∈𝒦. These probabilities are not all equal, meaning that some knowledge states might affect the general accuracy rate more than others. This is a rather useful consideration, because by listing all of the conditional probabilities $P(\hat{K}=K|K=K)$ across knowledge states one can easily discover states that will produce the least reliable assessments and those that will produce the most reliable ones. The higher the probability $P(\hat{K}=K|K=K)$, the higher the reliability of an assessment, if the true state is K. In this respect, a rather clear minimum requirement can be pointed out: The inequality

$$P(\hat{K}=K|K=K)\geq P(\hat{K}=R|K=K),$$

should hold for all knowledge states K∈𝒦 and all response patterns $R\in 2^{Q}$. This simply means that if the true state is K, then the outcome of an assessment should be K with maximum probability.

The case of total uncertainty (or maximum variance of the item response) is represented by the condition $\beta_{q}=\eta_{q}=1/2$ for all q∈Q. Under this condition, the marginal probability of observing a correct response to item q is

$$(1-\beta_{q})\pi_{q}+\eta_{q}(1-\pi_{q})=\frac{1}{2},$$

where $\pi_{q}=\sum_{K\in {𝒦}_{q}}\pi_{K}$ is the sum of probabilities of knowledge states containing item q. In this case, the conditional probability of R given K simplifies to

$$P(R|K)={\left(\frac{1}{2}\right)}^{\left|Q\right|},$$

and hence

$$P(K|R)=\frac{{\left(\frac{1}{2}\right)}^{\left|Q\right|}\pi_{K}}{\sum_{L\in 𝒦}{\left(\frac{1}{2}\right)}^{\left|Q\right|}\pi_{L}}=\pi_{K}.$$

Therefore,

$${𝒦}_{R}=\hat{𝒦}=\left\{K\in 𝒦:\pi_{K}=\underset{L\in 𝒦}{\max}\pi_{L}\right\},$$

for all $R\in 2^{Q}$, whereas

$${ℛ}_{K}=\left\{\begin{array}{cc} 2^{Q}\; & \text{if}\;K\in \hat{𝒦}\\ \emptyset \; & \text{if}\;K\in 𝒦\setminus \hat{𝒦}. \end{array}\right.$$

Thus, the probability $P(\hat{K}=K)$ is equal to

$$\begin{array}{ll} P_{0}(\hat{K}=K) & =\underset{K\in 𝒦}{\sum }\underset{R\in {ℛ}_{K}}{\sum }\frac{P(R|K)\pi_{K}}{\left|{𝒦}_{R}\right|}\;\\ & =\frac{1}{|\hat{𝒦}|2^{\left|Q\right|}}\underset{K\in 𝒦}{\sum }\underset{R\in {ℛ}_{K}}{\sum }\pi_{K}\;\\ & =\frac{1}{|\hat{𝒦}|2^{\left|Q\right|}}\underset{K\in 𝒦}{\sum }\pi_{K}|{ℛ}_{K}|\;\\ & =\frac{1}{|\hat{𝒦}|2^{\left|Q\right|}}\left(\underset{K\in \hat{𝒦}}{\sum }\pi_{K}|2^{Q}|+\underset{K\in 𝒦\setminus \hat{𝒦}}{\sum }\pi_{K}|\emptyset |\right)\;\\ & =\frac{1}{\left|\hat{𝒦}\right|}\underset{K\in \hat{𝒦}}{\sum }\pi_{K}.\; \end{array}$$

Then an index similar to Cohen's kappa is obtained as

(7) $$\begin{array}{ll} \kappa^{\prime } & =\frac{P(\hat{K}=K)-P_{0}(\hat{K}=K)}{1-P_{0}(\hat{K}=K)}\; \end{array}$$

(8) $$\begin{array}{ll} & =\frac{\sum_{K\in 𝒦}\sum_{R\in {ℛ}_{K}}\frac{P(R|K)\pi_{K}}{\left|{𝒦}_{R}\right|}-\frac{1}{\left|\hat{𝒦}\right|}\sum_{K\in \hat{𝒦}}\pi_{K}}{1-\frac{1}{\hat{𝒦}}\sum_{K\in \hat{𝒦}}\pi_{K}}.\; \end{array}$$

It is worth noticing that $\kappa^{\prime }$ tends to ρ as $P_{0}(\hat{K}=K)$ approaches zero. In turn, $P_{0}(\hat{K}=K)$ tends to zero as the cardinality of the knowledge structure increases, provided that only one modal state exists. Therefore, $\kappa^{\prime }$ is particularly interesting and useful when the cardinality of the knowledge structure is not large.

### 3.2 The expected discrepancy

The accuracy rate ρ informs about the probability that the estimated knowledge state $\hat{K}$ equals the true knowledge state K. However, in the case they are not equal, it says nothing about the discrepancy between the two. All in all, a not so high accuracy rate might be tolerated if, on the average, the difference between the estimated knowledge state $\hat{K}$ and the true one is small or negligible. An expected discrepancy between the true and modal state would be informative in this sense.

The symmetric difference between two subsets $A,B\in 2^{Q}$ is AΔB=(A∖B)∪(B∖A). The canonical distance between A and B is the cardinality |AΔB| of their symmetric difference. Given the two random variables K and $\hat{K}$, the new random variable $D=|K\Delta \hat{K}|$ is defined. For d∈D={0,1,…,|Q|}, let P(D=d) be the probability that the canonical distance between K and $\hat{K}$ equals d. It is easily seen that, $P(D=0)=P(K=\hat{K})$ is just the accuracy rate.

Now, for any knowledge state K∈𝒦 and any d∈D, define the collection

$${𝒦}_{d,K}=\{L\in 𝒦:|K\Delta L|=d\},$$

of all the knowledge states in 𝒦 having canonical distance d from K. Then, for any response pattern $R\in 2^{Q}$ and any knowledge state K∈𝒦 we have

$$P(D=d|K=K,R)=\frac{\left|{𝒦}_{R}\cap {𝒦}_{d,K}\right|}{\left|{𝒦}_{R}\right|}.$$

Therefore,

$$P(D=d)=\underset{K\in 𝒦}{\sum }\underset{R\in 2^{Q}}{\sum }\frac{\left|{𝒦}_{R}\cap {𝒦}_{d,K}\right|}{\left|{𝒦}_{R}\right|}P(R|K)\pi_{K}.$$

Then the expected discrepancy between the true state K and the modal state $\hat{K}$ is

(9) $$\begin{array}{ll} E(D) & =\underset{d\in D}{\sum }d\cdot P(D=d)\\ & =\underset{d\in D}{\sum }\underset{K\in 𝒦}{\sum }\underset{R\in 2^{Q}}{\sum }d\frac{\left|{𝒦}_{R}\cap {𝒦}_{d,K}\right|}{\left|{𝒦}_{R}\right|}P(R|K)\pi_{K}.\; \end{array}$$

### 3.3 Estimation of reliability indices from a finite data set

Both the expected accuracy rate ρ and the expected discrepancy E(D) are functions of the BLIM's parameters. As such, they can only be computed if the $\beta_{q}$, $\eta_{q}$ and $\pi_{K}$ parameters are known for all items q∈Q and all knowledge states K∈𝒦. Of course, in empirical applications of the BLIM the parameters at hand are usually unknown and need to be estimated from data. There exist at least two estimation methods: maximum likelihood (ML) (Stefanutti & Robusto, 2009) and minimum discrepancy (Heller & Wickelmaier, 2013). Thus, ρ and E(D) are unknown in practice; however, they can be estimated from data. If the true yet unknown values of $\beta_{q}$, $\eta_{q}$ and $\pi_{K}$ parameters are replaced by their ML estimates ${\hat{\beta }}_{q}$, ${\hat{\eta }}_{q}$ and ${\hat{\pi }}_{K}$ in Equations (6) and (9), then ML estimations of ρ and E(D) are obtained.

It should be noted that consistent ML estimates of ρ and E(D) can only be obtained if the BLIM is at least locally identifiable at the point $\hat{\theta }=(\hat{\beta },\hat{\eta },\hat{\pi })$ of the parameter space that maximizes the likelihood. Global and local identifiability of the BLIM have been thoroughly studied in the past (Heller, 2017; Spoto et al., 2012, 2013; Stefanutti et al., 2018), and it is well known that local identifiability depends on the particular knowledge structure to which the model is applied. Various solutions can be followed by the practitioner in the case in which the BLIM is applied to ‘non‐identifiable knowledge structures’. In Section 5 a complete example is developed, where one such solution is applied.

## 4 STUDY 1: ASYMPTOTIC BEHAVIOUR OF RELIABILITY INDICES

The asymptotic behaviour of the newly developed reliability indices was studied under different experimental conditions. A factorial design was employed, systematically manipulating the level of noise in the data, the number of test items, the number of knowledge states in the structure, and the shape of the probability distributions applied to the knowledge states. By varying these factors, we aimed to observe and analyse how the new indices behave across different scenarios.

Some expected results regarding the behaviour of the reliability indices based on the manipulated variables are:

1. As the noise level in the data increases, reliability decreases indicating a deterioration in the assessment accuracy. Specifically, the expected accuracy rate should decrease in value under high‐noise conditions, while the mean of the discrepancy distribution should increase.
2. Increasing the number of test items should enhance the reliability, as a larger item pool generally provides more information, reflecting more robust measurement. The estimated accuracy rate is likely to yield higher values with an increased number of items. In comparison, the expected values of the estimated discrepancy distribution should provide smaller values.
3. A higher number of knowledge states might challenge the reliability, potentially resulting in decreased values of the estimated accuracy rate when the structure becomes more complex and increased values of the mean of the discrepancy distribution.
4. The probability distribution on the knowledge states is likely to affect the reliability. For instance, under low item error conditions a uniform distribution across knowledge states may result in lower reliability compared to a non‐uniform one.

Overall, this study allows us to gain insight into the behaviour of the indices under diverse and potentially challenging conditions that qualitatively represent more or less reliable situations. Thus, the study provides information about the practical utility of these indices useful for interpreting results of empirical applications.

### 4.1 Simulation design and data set generation

The four variables manipulated for testing the reliability were: (1) number of test items; (2) number of knowledge states in the structure; (3) shape of the probability distribution on the knowledge states; and (4) item error probabilities. A detailed description of each of these variables follows.

Three different domains $Q_{1}$, $Q_{2}$ and $Q_{3}$ were considered with cardinalities 10, 15 and 20, respectively. These cardinalities correspond to different plausible configurations for the number of test items, spanning from a small to a moderately sized set. For each of these three domains, six different knowledge structures were constructed, giving rise to a total number of 18 knowledge structures. Each of the 18 structures is denoted by ${𝒦}_{ij}$, where i∈{1,2,3} indicates the domain $Q_{i}$, and j∈{1,2,…,6} indicates the size of the structure. The first two structures ${𝒦}_{i1}$ and ${𝒦}_{i2}$ are particularly noteworthy. Indeed, structures ${𝒦}_{i1}=\{\emptyset ,Q_{i}\}$, for i∈{1,2,3}, contain only the two states where individuals master all or nothing. This setup aligns with the assumptions of CTT, in which all items are intended to measure the same construct. Similarly, all the structures ${𝒦}_{i2}$ are unidimensional and include $|Q_{i}|+1$ states, reflecting a linear order on the items. These structures represent unidimensional tests where items vary in difficulty and remain aligned along a single dimension. For each of the domains, the remaining four structures represent more complex, multidimensional scenarios, with a number of knowledge states equal to 30, 70, 100 and 200, respectively, for structures ${𝒦}_{i3}$, ${𝒦}_{i4}$, ${𝒦}_{i5}$ and ${𝒦}_{i6}$. These cardinalities were selected to represent a broad range of structure sizes, from relatively small to moderately large knowledge structures. This variation allows us to examine how the reliability index behaves across different levels of granularity in the representation of knowledge, reflecting realistic applications that range from simpler to more complex assessment scenarios.

Each of the 18 knowledge structures was constructed by first taking the empty set ∅ and the total set $Q_{i}$, and then adding a collection of intermediate subsets of knowledge states. These intermediate subsets, were obtained by randomly sampling, without replacement, from the power set of $Q_{i}$, excluding the empty set and the total set.

Five different shapes were considered for the probability distributions on the knowledge states. In the ‘uniform’ distributions, all knowledge states have an equal probability (i.e. $1/|{𝒦}_{ij}|$). For each of the ‘random’ distributions, first a number $\left|{𝒦}_{ij}\right|$ of uniformly distributed random values in the interval (0,1) was generated. Then, the obtained values were normalized to sum up to 1.

In the ‘random symmetric’ distributions, probabilities were generated by sampling from a binomial distribution. Specifically, the number of successes was the cardinality of each knowledge state, while the number of trials varied from 0 to $\left|{𝒦}_{ij}\right|$. The success probability was set to .50 to ensure symmetry. Once the probability distribution was generated, individual probability values were assigned to each knowledge state based on its cardinality. Lower probabilities were allocated to states with either low or high cardinalities, while higher probabilities were assigned to states with medium cardinalities (approximately half of the total number of items).

The ‘random non‐uniform’, the ‘random right‐asymmetric’ and the ‘random left‐asymmetric’ probability distributions were drawn from a $\left|{𝒦}_{ij}\right|$‐dimensional Dirichlet distribution, with parameters α=1. This parameter value produces a Dirichlet distribution that is uniform across the simplex of all the $\left|{𝒦}_{ij}\right|$‐dimensional points, each representing a unique probability distribution over the set ${𝒦}_{ij}$ of knowledge states. The extracted probability distribution was used in the random non‐uniform condition. The same procedure was used for generating the distributions of random left‐asymmetric and random right‐asymmetric conditions. The only difference was that, after generating the distributions, the probability values were sorted in ascending order for the random left‐asymmetric condition and in descending order for the random right‐asymmetric one. Each ordered probability value was then assigned to a knowledge state according to its cardinality. In the random left‐asymmetric distribution, lower probabilities were assigned to states with smaller cardinalities, while higher probabilities were assigned to states with larger cardinalities. Conversely, in the random right‐asymmetric distribution, lower probabilities were allocated to states with higher cardinalities, while higher probabilities were assigned to states with smaller cardinalities.

Finally, the item error probability was manipulated through the false negative $\beta_{q}$ and the false positive $\eta_{q}$ probabilities of the BLIM. Specifically, for each item q∈Q, these parameters were randomly sampled from a uniform distribution over 100 intervals within the range (0,a), where a varied from .01 to 1, with increments of .01. Moreover, the additional constraint β+η<1 was assured.

This factorial design results in a total of 9000 simulation conditions (5 distribution shapes × 6 knowledge structure cardinalities × 3 numbers of items × 100 error levels). The following dependencies among the factors exist. The error parameters vary only with the number of items and they are independent from all the other factors. The number of states depends on the number of items only in the special case where ${𝒦}_{ij}$ is a linear order. The probability distribution on the knowledge states only depends on the shape of the distribution and on the cardinality of the structure. When one of the factors changes another element of this design changes too only if there is a dependency towards it.

### 4.2 Methods

For each of the 9000 conditions, the estimated accuracy rate $\hat{\rho }$, the expected value of the discrepancy distribution of the distance $D({𝒦}_{ij},{\hat{𝒦}}_{ij})$, and the ‘pseudo’ Cohen's $\kappa^{\prime }$ were computed by using Equations (6), (9), and (7), respectively. Then, average values of these indices were computed as follows. In order to summarize the results on the amount of error in the data, the 100 different conditions were partitioned in 10 classes each corresponding to a sub‐interval of length .10 of the entire interval [0,1). Then, average values of the estimated accuracy rate ρ and of the ‘pseudo’ Cohen's $\kappa^{\prime }$ were computed in each interval.

The MATLAB functions that implement the algorithms for estimating both the accuracy rate $\hat{\rho }$ and the expected value of the discrepancy distribution E(D) are available at the link https://osf.io/nr2ug/overview.

### 4.3 Results: Accuracy rate and expected discrepancy

Figure 1 shows the results about the accuracy rate (solid lines) and the expected discrepancy (dashed lines) across varying levels of noise (x‐axis), when the knowledge state probability distribution is uniform. Each panel corresponds to a different cardinality of the knowledge structure, ranging from 2 to 200. The results are shown for domains with three different item numbers: $|Q_{i}|=10$ (blue lines), $|Q_{i}|=15$ (orange lines) and $|Q_{i}|=20$ (yellow lines).

**FIGURE 1:**
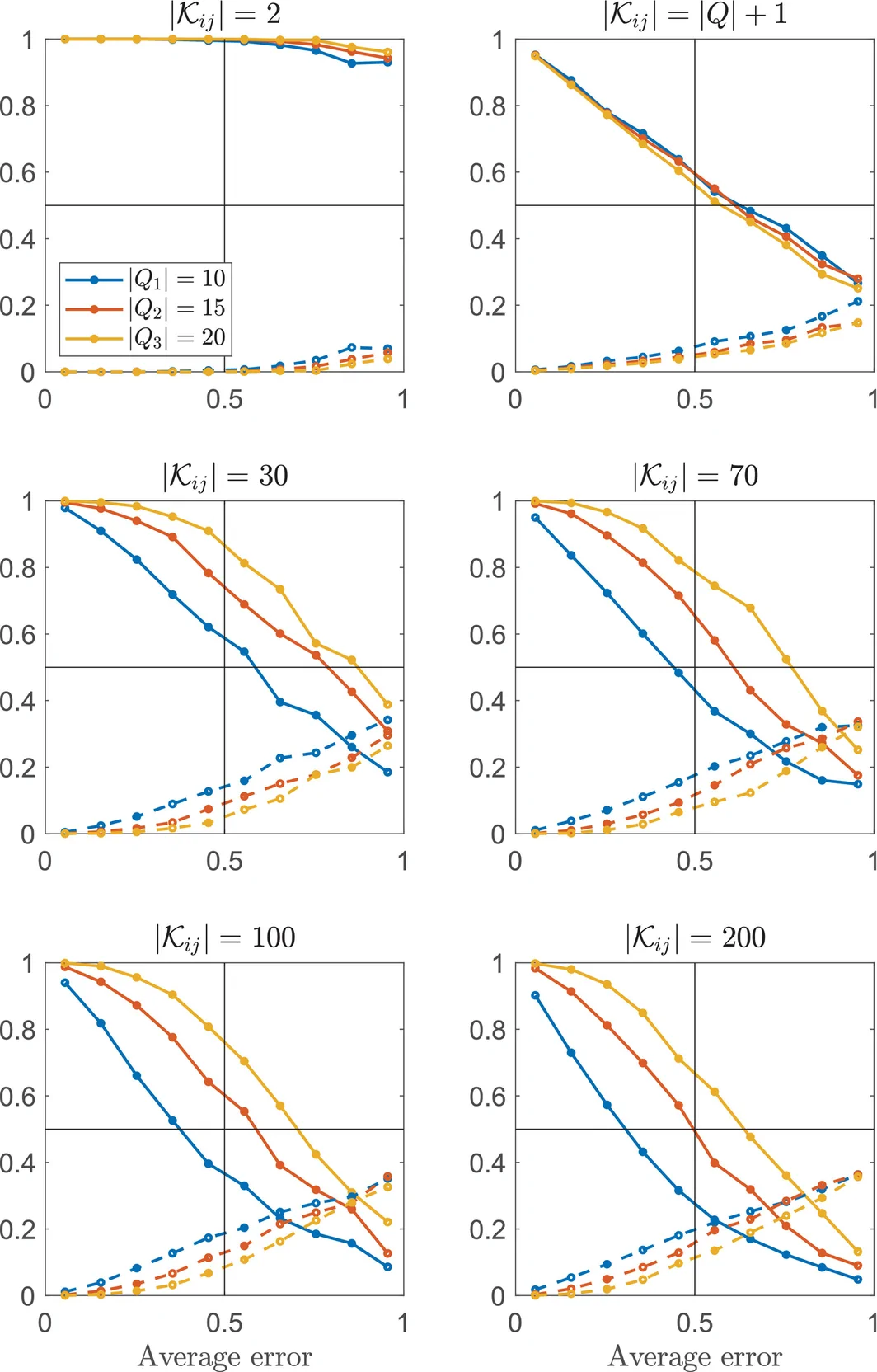
Comparison of accuracy rate and expected discrepancy across different cardinalities of the knowledge structure ($\left|K_{ij}\right|$) and noise levels. The panels display results for varying cardinalities of the knowledge structure. The blue, orange and yellow lines correspond to different number of items $|Q_{i}|=10$, 15 and 20, respectively. The accuracy rate (solid lines) and expected discrepancy (dashed) are plotted as a function of the average error (x‐axis).

Across all panels, the accuracy rate decreased as the average error increases. For cardinalities $|{𝒦}_{ij}|\geq 30$, the decline in accuracy rate was more pronounced, particularly for smaller domains ($|Q_{i}|=10$). In general, higher domain cardinalities ($|Q_{i}|=20$) were associated with higher accuracy rates across all noise levels. On the contrary, the expected discrepancy increased with the average error. The effect was more evident for larger item sets ($|Q_{i}|=15$ and $|Q_{i}|=20$) compared to the smaller one. For smaller cardinalities of $\left|{𝒦}_{ij}\right|$ (i.e. 2 and |Q|+1), the performances of the two indices seemed not to be affected by the number of items as it can be seen from the two top panels of the figure. Things were different for what concerns larger cardinalities where number of items seemed to have an effect on the two indices. In particular, the higher the number of items the better the performance, as shown in the middle and bottom panels of the figure.

In Figure 2 the same two metrics (the accuracy rate on the left and expected discrepancy on the right) are now organized to compare more directly the results for different cardinalities of the structure by fixing the number of items. Each row of the figure displays the results with a different number of items.

**FIGURE 2:**
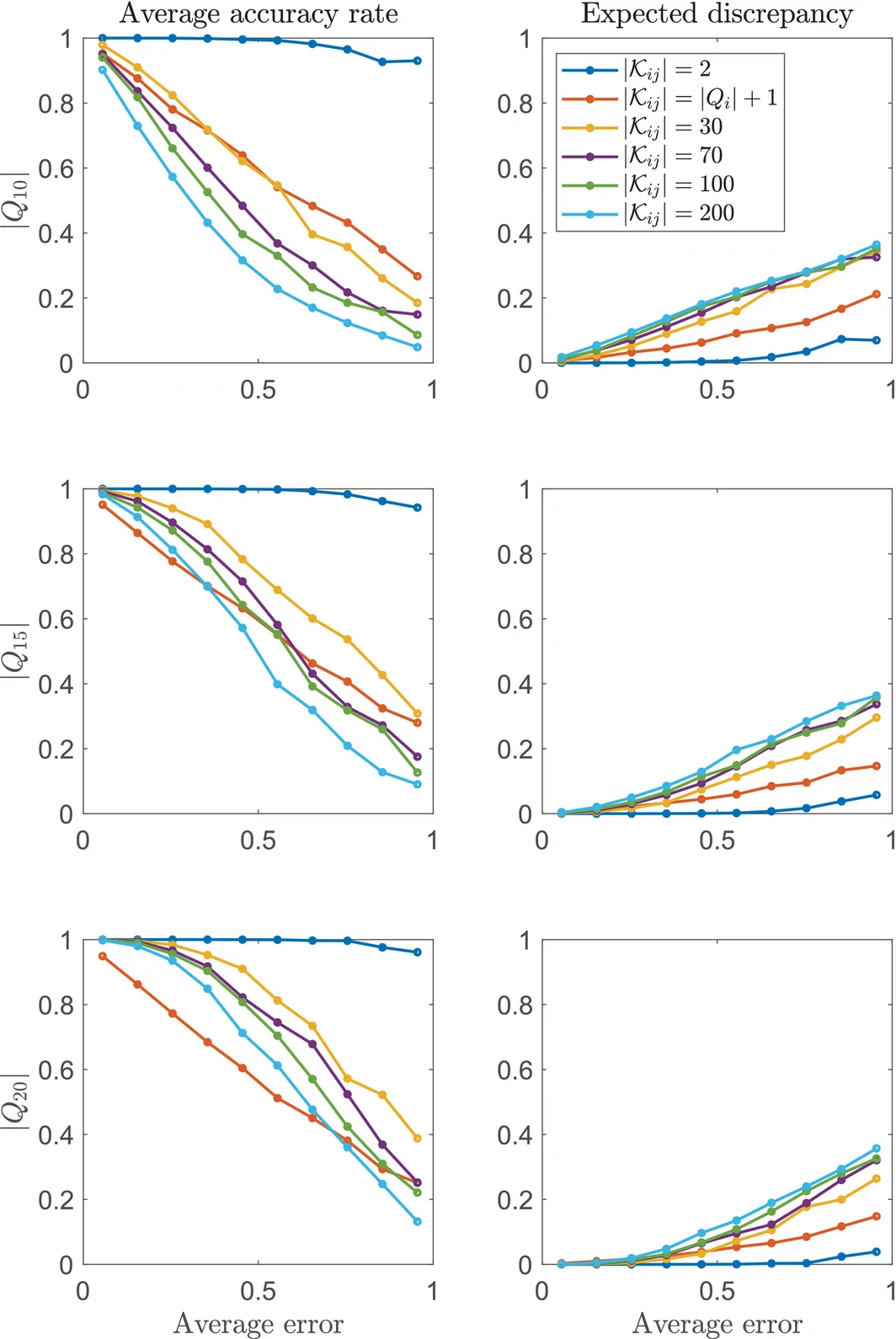
Comparison of accuracy rate and expected discrepancy across different cardinalities of the knowledge structure ($\left|K_{j}\right|$) and noise levels. The panels display results for varying numbers of items in the domain (|Q|=10, 15, 20). The accuracy rate (on the left) and expected discrepancy (on the right) are plotted as a function of the average error (x‐axis).

Results indicated a trade‐off between the number of items and the cardinality of the knowledge structure. For low cardinalities (e.g. $|K_{ij}|=2$ or $|K_{ij}|=|Q_{i}|+1$), accuracy remained stable regardless of the number of items, suggesting that simpler structures were less affected by the increase in items. In contrast, in case of higher cardinalities (e.g. $|K_{ij}|=100$ or $|K_{ij}|=200$) having more items could be beneficial to accuracy, as they provide additional information. This suggests that for high‐cardinality structures, increasing the number of items can compensate for accuracy loss.

The results for the accuracy rate and the discrepancy, when the distribution on knowledge states was ‘random non‐uniform’, ‘random right‐asymmetric’ or ‘random left‐asymmetric’ were overall very similar to those observed with a uniform distribution (see Figures S1–S3 and S5–S7). The only notable difference was that, the accuracy rate was systematically higher and the discrepancy was worse under the non‐uniform distributions rather than the uniform one. This was an expected outcome because in a non‐uniform distribution some states were more likely than others. When the distribution was ‘random symmetric’, the results remained very similar to those obtained under all the other non‐uniform distributions, with the key difference being that overall performance further improved (see Figures S4 and S8).

To better understand the effect of the distribution on the accuracy rate and specifically to determine whether the observed differences were due to the shape of the distribution or to other factors, Shannon's entropy of all distributions was calculated. By analysing the entropy, we aimed to disentangle whether the performance differences stem from the structural characteristics of the distribution itself or from the inherent complexity introduced by varying levels of uncertainty. Figure 3 shows the variation of Shannon's entropy as a function of the number of knowledge states, for the three domains $|Q_{i}|=10$, $|Q_{i}|=15$ and $|Q_{i}|=20$ (panels form the left to the right, respectively). The yellow line represents the symmetric probability distribution, while the blue one corresponds to the uniform distribution. The remaining lines depict results for the three random probability distributions.

**FIGURE 3:**
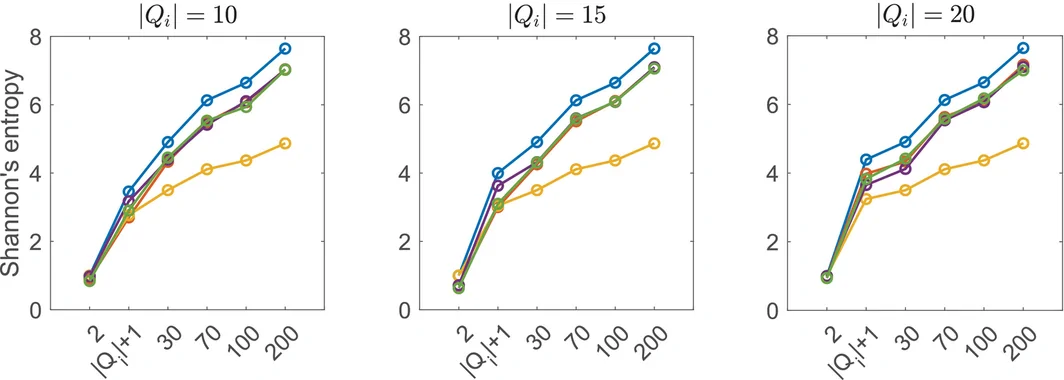
Shannon entropy as a function of the number of knowledge states for the three domains $|Q_{i}|=10$ (left panel), $|Q_{i}|=15$ (mid panel) and $|Q_{i}|=20$ (right panel). The blue line represents the uniform probability distribution, while the yellow line corresponds to the symmetric distribution. The remaining lines represent results for the three random probability distributions.

Across all panels, Shannon's entropy increased with the number of knowledge states, as expected. The uniform distribution consistently exhibited the highest entropy values, reflecting its maximum uncertainty. Conversely, the symmetric distribution demonstrated lower entropy levels, indicating less uncertainty. The random distributions fell between these two extremes. These results are in line with those obtained for the accuracy rate, where the best performance was observed for the symmetric distribution and the worse performance was observed for the uniform distribution. The totally ordered structures deserve a separate comment. With 20 items, the accuracy rates for these structures were worse than those of the others structures, although the former have a lower entropy than the latter.

### 4.4 Results: Accuracy rate and KS‐Reliability comparison

Figure 4 compares KS‐reliability with accuracy rate across different knowledge structure cardinalities (panels in the figure) and numbers of items (the three coloured lines).

**FIGURE 4:**
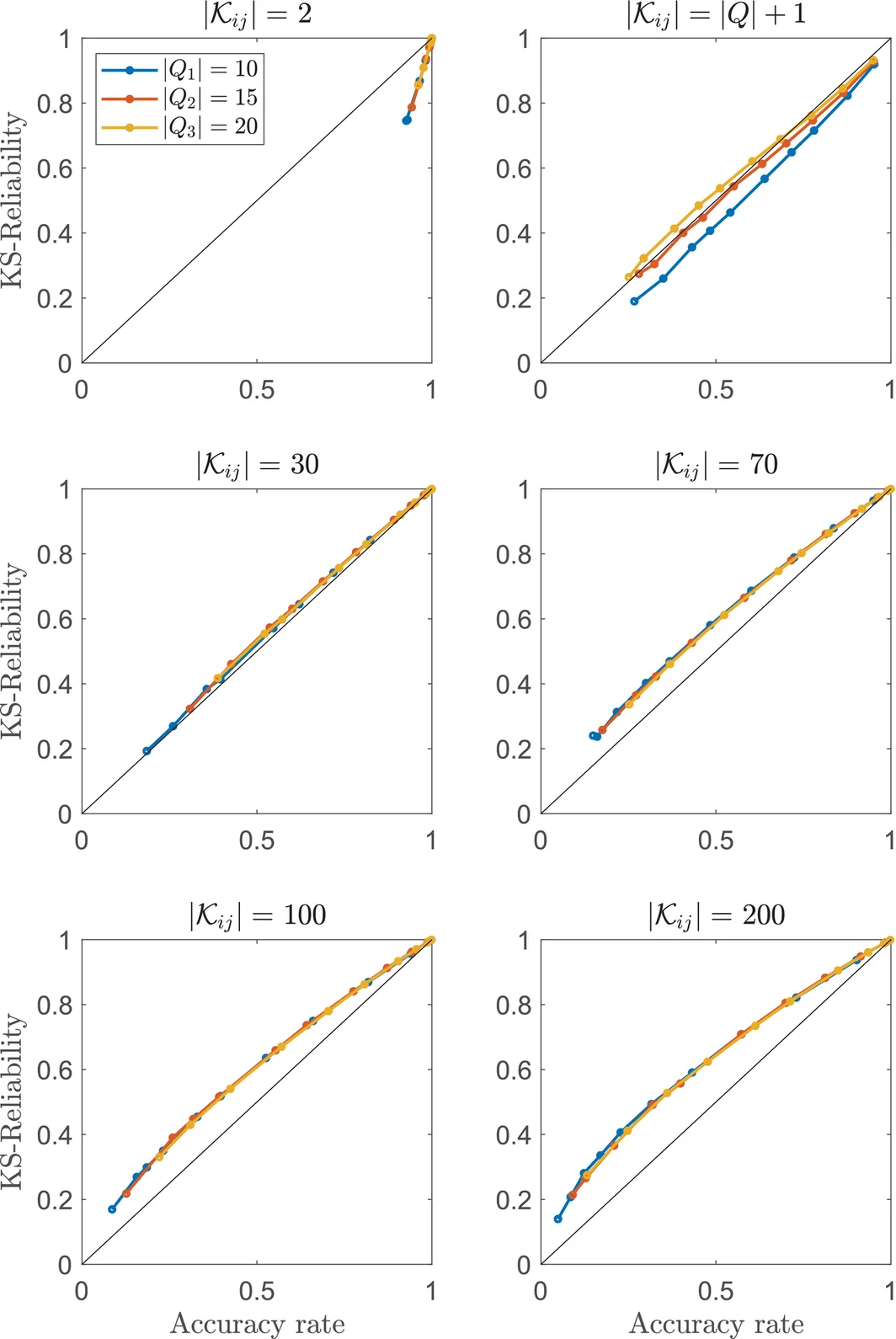
Comparison between KS‐Reliability and accuracy rate estimates across different knowledge structure cardinalities (panels in the figure) and numbers of items (the three coloured lines).

Overall, KS‐reliability closely aligned with the accuracy rate across all conditions, as highlighted by points scattered close to the diagonal line (x=y). In some cases the relation between the two was linear (i.e. $|{𝒦}_{ij}|=2$, $|{𝒦}_{ij}|=|Q_{i}|+1$ and $|{𝒦}_{ij}|=30$) and it approximated the identity for cardinalities $|{𝒦}_{ij}|=|Q_{i}|+1$ and $|{𝒦}_{ij}|=30$. When the cardinality of the structure increased, the relation between the two metrics became a concave and monotone increasing function. Similar results were obtained for the other knowledge state probability distributions (see Figures S9–S12).

To better understand the relation between the two metrics, one should consider what each represents. Of the total information required to fully describe the knowledge state K, the KS‐Reliability index is the proportion of information captured by the observed response pattern R. In contrast, the accuracy rate is the probability that K is correctly recovered during an assessment. This study showed a monotonically increasing relationship between KS‐Reliability and accuracy rate. As expected, greater amounts of information is associated with improved predictive capability, although the relationship between the two variables is not linear.

## 5 STUDY 2: FINITE SAMPLE BEHAVIOUR OF RELIABILITY INDICES

While the previous study focused on evaluating the theoretical properties of the proposed reliability indices through large‐scale simulations, the present study extends this investigation by considering finite sample scenarios. Examining the behaviour of the indices in finite samples allows us to assess its robustness and practical applicability under realistic testing conditions.

A key distinction between this study and the previous one lies in the treatment of the probability distribution over knowledge states. In the previous study, reliability was examined as a function of the theoretical probability distribution over all possible knowledge states. Here, instead, we maintain a fixed probability distribution (a random non‐uniform) but introduce sample size as a manipulated variable. This allows us to evaluate how estimation accuracy and index stability vary as a function of sample size, a crucial consideration for practitioners working with limited data.

### 5.1 Simulation design and data set generation

To assess the properties of the newly proposed reliability index, we conducted a simulation in which a subset of the variables manipulated in the previous study was selected. This selection was necessary for computational reasons, ensuring that the simulations remained feasible while still capturing the key factors influencing reliability.

The three domains $Q_{1}$, $Q_{2}$ and $Q_{3}$ of the previous study were maintained. Instead, of the 18 knowledge structures of the previous study, 12 were selected and used for running simulations, that is ${𝒦}_{i1}$, ${𝒦}_{i2}$, ${𝒦}_{i4}$ and ${𝒦}_{i6}$, representing, respectively, structures with cardinalities 2, $|Q_{i}|+1$, 70 and 200.

An important issue that must be addressed when applying a probabilistic model to a finite sample is the identifiability of its parameters. The model used in this study is the BLIM, whose identifiability has been thoroughly studied (Heller, 2017; Spoto et al., 2012, 2013; Stefanutti et al., 2018). Among others, a well‐known problem is that there are two large classes of knowledge structures for which the BLIM is known to be non‐identifiable. These are called the forward‐graded (FG) and backward‐graded (BG) knowledge structures (Spoto et al., 2013). A knowledge structure 𝒦 on a set Q of items is backward‐graded in an item q∈Q if K∖{q} belongs to 𝒦 for every knowledge state K∈𝒦. Conversely, the knowledge structure 𝒦 is forward‐graded in q if K∪{q} belongs to 𝒦 for every knowledge state K∈𝒦 (Spoto et al., 2013).

The knowledge structures used in the simulation conditions, where the cardinality is $|Q_{i}|+1$ (representing the total order relation among the items), are both FG and BG—specifically, they are FG in the easiest item and BG in the most difficult item. However, adding an equally informative item for each of the items involved in forward or backward gradedness makes the structure neither FG nor BG anymore. Therefore, any identifiability issue related to BG and FG are resolved (Spoto et al., 2013). Therefore, an equally informative item was introduced for both the easiest and most difficult items in each domain $Q_{i}$ when structure ${𝒦}_{i2}$ was considered.

Concerning the item error probability, the type of manipulation was the same as the one of the previous study with a different number of the intervals. Specifically, for each item $q\in Q_{i}$, these parameters were randomly sampled from a uniform distribution over three intervals within the range (0,a), where a∈{.10,.20,.40}. With these intervals, the constraint $\beta_{q}+\eta_{q}<1$ is always assured.

Finally, the sample size was set to 100, 500 or 1000. By crossing the four different variables, a number of 3×4×3×3=108 different experimental conditions (Table 1) were obtained, ensuring a broad exploration of how different situations might affect the behavior of the reliability indices.

**TABLE 1:** The four manipulated variables that lead to the 3×4×3×3=108 experimental conditions of Simulation Study 2.

Domain		Structure		Error		Sample size
$\left\{\begin{array}{c} Q_{1}\\ Q_{2}\\ Q_{3} \end{array}\right\}$	×	$\left\{\begin{array}{c} {𝒦}_{i1}\\ {𝒦}_{i2}\\ {𝒦}_{i4}\\ {𝒦}_{i6} \end{array}\right\}$	×	$\left\{\begin{array}{c} .10\\ .20\\ .40 \end{array}\right\}$	×	$\left\{\begin{array}{c} 100\\ 500\\ 1000 \end{array}\right\}$

In all these conditions, the probability distribution among the knowledge states was kept constant and it was the ‘random non‐uniform distribution’ used in the previous study. For each of the 108 simulation conditions, 100 samples were simulated by using the BLIM as the generator model.

### 5.2 Methods

For each of the 108×100=10,800 simulated samples, the estimated accuracy rate ρ, the expected value $E(|K\Delta \hat{K}|)$ of the discrepancy distribution, the ‘pseudo’ Cohen's $\kappa^{\prime }$, and the KS‐Reliability indices were estimated. Then, the average bias and the corresponding standard error were computed for each index. More precisely, the estimated bias for each index $\theta \in \{\rho ,\kappa^{\prime },E(|K\Delta \hat{K}|),\text{KS‐Reliability}\}$ was computed as follows:

(10) $$\text{bias}(\hat{\theta })=\bar{\theta }-\theta ,$$

where: θ is the true value of the index computed with the parameter values used for generating the data and $\bar{\theta }$ is the arithmetic mean of the 100 estimation of the θ index. The standard error of each θ index was estimated as follows:

(11) $$\sigma (\hat{\theta })=\sqrt{\frac{1}{100}\overset{100}{\underset{k=1}{\sum }}{\left({\hat{\theta }}_{k}-\bar{\theta }\right)}^{2}}.$$

All simulations and data analyses based on the BLIM were run by using the MATLAB ‘kst’ toolbox described in Brancaccio et al. (2024) and available at https://it.mathworks.com/matlabcentral/fileexchange/157751‐kst‐toolbox.

### 5.3 Results

Figure 5 shows the estimation bias of the four indices as a function of the amount of error in the data (the four top panels) and of the true values of the indices (the four bottom panels). In all panels, each curve corresponds to a different cardinality of the knowledge structure, as indicated in the legend (i.e. $2,|Q_{i}|+1,70,200$).

**FIGURE 5:**
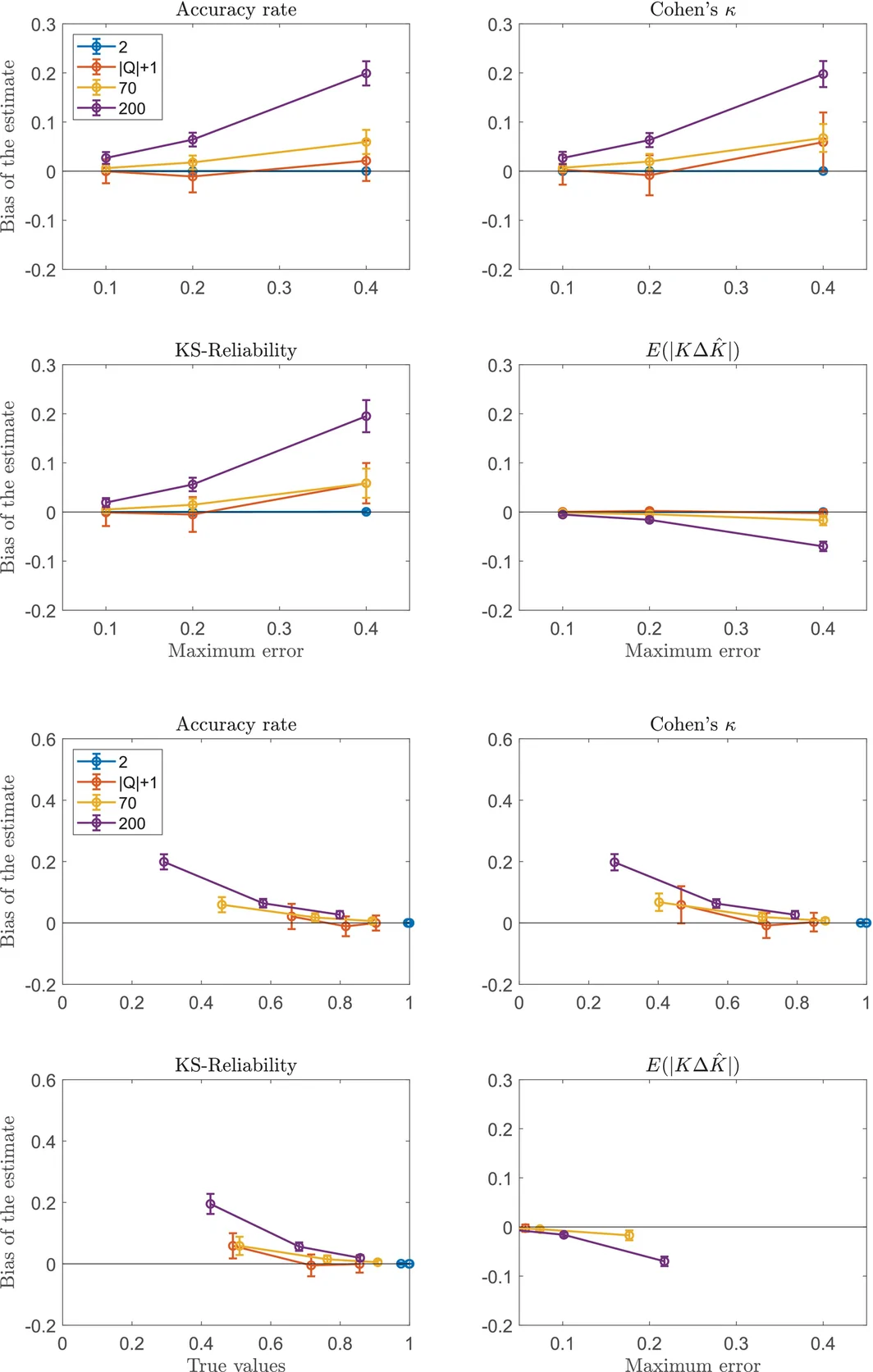
Bias of reliability indices as a function of the manipulated error (the four panels to the top) and as a function of the true value of the indices (the four panels to the bottom).

Concerning the four top panels of the figure, a systematic trend was observed across all indices: as the error increased, the bias monotonically increased, with variations depending on the cardinality of the structure. When cardinality is 2, the bias remained relatively close to zero, whereas as cardinality increases, the bias tended to increase too. A different comment is deserved by the index $E(|K\Delta \hat{K}|)$ at the bottom‐right panel, which is the expected distance between the estimated and true states. In this case, the distance increased as the amount of error in the data increased, though it never exceeds 10% of the total number of items. This indicates that even under increasing error conditions, the estimation remains reasonably accurate. It should be observed that the bias here is an underestimation. This is in line with the overestimation of the accuracy rate indices.

Concerning the four bottom panels of the figure, the key observation is that the bias was not constant across the true reliability values. For lower true reliability levels, the bias appeared more pronounced, approaching an overestimation of about .20 when cardinality is 200. As true reliability increased, the bias generally decreased for all indices, approaching zero. Results on the expected distance $E(|K\Delta \hat{K}|)$ showed an underestimation that tended to increase as the true value increases.

## 6 STUDY 3: EMPIRICAL EXAMPLE

To illustrate how the ρ accuracy rate and the expected discrepancy can be applied and interpreted in empirical studies, we used a real data set. The selection of the data set followed three criteria: (i) The data set must have previously been used to validate a knowledge structure using the BLIM; (ii) The model must be identifiable; (iii) The data set must be publicly available for research purposes.

We selected data set dataDS.mat, which is included in the kst MATLAB toolbox (Brancaccio et al., 2024). This data set, denoted here as 𝒟, was collected by de Chiusole et al. (2018) and consists of the responses of 286 Italian psychology university students to 24 questions on the domain of descriptive statistics.

A key feature of this data set is that the $\left|Q_{DS}\right|=24$ items were generated automatically from 12 ‘item templates,’ resulting in 12 pairs of equally informative items (two instances q,r∈Q of an item template are equivalent if and only if they belong to exactly the same states in 𝒦). Readers can refer to de Chiusole et al. (2018, table 2) for further details on the test items and the procedure used to generate them. The presence of pairs of equally informative items is particularly important because it provides an identifiable knowledge structure, in most cases. Indeed, Spoto et al. (2013) formulated a conjecture stating that if (Q,𝒦) is the extension of a knowledge structure to include equally informative items, then the BLIM is locally identifiable for (Q,𝒦). The knowledge structure ${𝒦}_{DS}$ for this domain was delineated using the skill function functionSD.mat, available in the kst MATLAB toolbox and displayed in Table 3 of de Chiusole et al. (2018).

With a test structure that leads to an identifiable knowledge structure, the focus shifted to evaluating the goodness‐of‐fit of the BLIM to the data and the computational feasibility of the accuracy rate indices. Notably, the algorithms developed for computing estimates of both the accuracy rate ρ and the KS‐Reliability indices require deriving the entire power set $2^{Q}$. In the case of $Q_{DS}$, this power set consists of $2^{24}=16,777,216$ subsets, which is computationally infeasible on standard computers. Therefore, we selected a subset of item templates from $Q_{DS}$ to obtain a more manageable knowledge domain. Specifically, we chose eight item templates, corresponding to 16 items. Item templates 2–9 were included, while item template 1 was excluded because it contained item pairs that were not equally informative, as explained in de Chiusole et al. (2018).

To obtain a reduced version of ${𝒦}_{DS}$ and the data set 𝒟, we selected only items 2–9 and their corresponding equally informative items 14–21 for all knowledge states in 𝒦 and response patterns in 𝒟. The resulting collection of knowledge states was then filtered to remove duplicate states, yielding a knowledge structure ${𝒦}_{DS}^{\prime }$ consisting of 60 states. An application of the BLIMIT function (Stefanutti et al., 2012) to this knowledge structure, now defined over 16 items, confirmed its identifiability.

The subsequent analysis consisted in fitting the BLIM based on ${𝒦}_{DS}^{\prime }$ to the reduced data set ${𝒟}^{\prime }$ and estimating the ρ accuracy rate, the expected discrepancy distribution $E(|K\Delta \hat{K}|)$ and KS‐Reliability. The results indicated a good fit of the BLIM to the data (*p*‐value = .41) and a high reliability of the test, as estimated by both ρ=.87 and KS‐Reliability (.86). Regarding the discrepancy distribution, the probability of $|K\Delta \hat{K}|=2$ was .11, the probability of $|K\Delta \hat{K}|=4$ was .01, and the probability of $|K\Delta \hat{K}|\geq 6$ was less than .001. It is worth noting that, in this case, the observable distances can only be multiples of 2 up to 16 (the number of items). This aspect arises from the equality constraint imposed on the pairs of items generated from the same item template. Overall, the expected discrepancy turned out to be E(D)=1.32 items of 16 (about 8%).

These findings suggest that the proposed indices can effectively quantify the reliability of KST‐based assessments. Furthermore, the analysis of the expected discrepancy distribution provides additional insights. The fact that small discrepancies occur with relatively higher probability, while larger discrepancies are extremely rare supports the validity of the estimated knowledge states.

## 7 FINAL REMARKS

In this article we analytically derived, study and test three new reliability indices for KST obtained from the in depth analysis of the probability distribution of the discrepancy between the true and estimated knowledge states. These indices are the *accuracy rate* ρ, the pseudo Cohen's $\kappa^{\prime }$ and the *expected discrepancy*. The first is the probability $\rho =P(K=\hat{K})$ that the modal state $\hat{K}$ resulting from an assessment is equal to the true latent knowledge state of an individual. The second provides information—especially in relatively small structures—about the incremental reliability obtained from ρ over the case of total uncertainty. Finally, the expected discrepancy E(D), informs about the expected distance between the estimated and the true knowledge state.

Compared to the reliability indices proposed in de Chiusole et al. (2024), in particular the KS‐Reliability index representing an indirect measure of assessment accuracy, the newly proposed indices present the advantage of being immediately interpretable either like probabilities or proportions (ρ and $\kappa^{\prime }$) or discrepancies. In this sense they can be conceived as a direct estimation, obtained starting form the parameters of the model, of the True Positive Rate (TPR), used in de Chiusole et al. (2024) to evaluate the behaviour of KS‐Reliability, but not computable from observed data and, therefore, not usable in real world applications.

The performance of the proposed indices was examined in two simulation studies: The first focused on their asymptotic behaviour (Study 1); the second tested them in finite sample conditions (Study 2).

Study 1 revealed a trade‐off between the number of items and the cardinality of the knowledge structure in reliability measurement. In fact, while no particular differences in accuracy are observed in low cardinality structures (e.g. $|K_{ij}|=2$ or $|K_{ij}|=|Q_{i}|+1$) at different error levels, increasing differences are observed for high cardinality structures (e.g. $|K_{ij}|=100$ or $|K_{ij}|=200$). In particular, for these last the higher the number of items, the better the accuracy of the assessment. This could be explained by the fact that the presence of more items provides additional information for the reconstruction of the true state compensating the reduction in accuracy due to the error level.

Considering the results of Study 2, it can be argued, once again, that the detrimental effect of the amount of error on the quality of the estimation of reliability tends to be stronger when larger structures are taken into account. In particular, this effect seems to result in an greater overestimation of the true reliability. This overestimation becomes more pronounced when the error exceeds .20 and the structure cardinality increases. Altogether, this evidence suggests to carefully take into account the role of the cardinality when interpreting reliability measures.

Additional insights can be drawn from the last four panels of Figure 5. The results suggest the existence of a reliability range—between .70 and 1.00 for ρ, $\kappa^{\prime }$, and KS‐Reliability—that remains unbiased and accurate regardless of structure cardinality, item count and error magnitude. However, estimated values around .60 require more cautious interpretation, particularly in larger structures, as overestimation effects may make the reliability measure less trustworthy. In such cases, examining the error parameters $\eta_{q}$ and $\beta_{q}$ could help identify true reliability levels. Specifically, if the estimated reliability value varies significantly when items with the highest error parameters are removed, the original reliability estimate is likely overestimated. Conversely, stability in the index suggests a more precise estimation. Following this reasoning, it may be beneficial to compute the reliability index ρ separately for each item in the domain q∈Q. The result would be an index computed by taking into account only the parameters $\eta_{q}$ and $\beta_{q}$ of that item and the marginal probability of q. This operation could provide further information, similarly the one obtained in Classical Test Theory when overall indices (e.g. Cronbach's α) are reported together with item‐specific information like the *item‐rest correlation* or the α value computed by removing an item at a time.

Finally, the empirical example demonstrates how the newly introduced indices contribute to a more comprehensive evaluation of test reliability within the KST framework. Future research could explore their application to different knowledge domains and investigate their sensitivity to variations in test length and item dependencies.

In conclusion, both the simulation studies and the empirical example suggest that, despite some minor points and some cautions in the interpretation of values in critical situations, the considered reliability indices provide highly accurate estimates and could represent a valuable contribution in KST reliability measurement.

Traditionally, the concepts of reliability and measurement error are closely tied to the consistency of scores across multiple parallel assessments, as happens in CTT and IRT. In CTT and in many IRT models, this is required because there is no way to separate the error variance component from that of the true score. In KST, parallel assessments are not necessary for arriving at a measure of reliability. Indeed, theoretical assumptions behind the BLIM allow to estimate the item related error parameters, which provide the basis for a direct estimation of a reliability index. This is because in KST the notion of a true and latent knowledge state is separate from the observed response pattern.

Many reliability indices in the classical psychometric literature are based on repeated assessments, like for instance, test‐retest and parallel forms. An interesting question is if the indices proposed in this paper can receive an interpretation related to these classical reliability indices. A possible way is as follows.

Suppose a group of individuals are assessed at two different time points with the same set Q of items. Let ${\hat{K}}_{1}$ and ${\hat{K}}_{2}$ be random variables representing, respectively, the knowledge states estimated at time 1 and time 2. If the time interval between the two assessments is relatively short, in a reliable test one expects a high probability of observing identity between ${\hat{K}}_{1}$ and ${\hat{K}}_{2}$. Hence, the probability $P({\hat{K}}_{1}={\hat{K}}_{2})$ can be regarded as a measure of test‐retest reliability in KST. Under the usual local independence assumptions, and the specific assumption that the true, but unknown knowledge state remains stable across the two time points, one can write (let K∈𝒦 represent the true state):

$$\begin{array}{l} P({\hat{K}}_{1}={\hat{K}}_{2})=\underset{K\in 𝒦}{\sum }P({\hat{K}}_{1}={\hat{K}}_{2}|K)\pi_{K}, \end{array}$$

where

$$\begin{array}{ll} P({\hat{K}}_{1}={\hat{K}}_{2}|K) & =\underset{L\in 𝒦}{\sum }P({\hat{K}}_{1}=L,{\hat{K}}_{2}=L|K)\;\\ & =\underset{L\in 𝒦}{\sum }P({\hat{K}}_{1}=L|K)P({\hat{K}}_{2}=L|K).\; \end{array}$$

By plugging the right hand side of this last equation into the previous one, one obtains

$$P({\hat{K}}_{1}={\hat{K}}_{2})=\underset{K\in 𝒦}{\sum }\underset{L\in 𝒦}{\sum }P({\hat{K}}_{1}=L|K)P({\hat{K}}_{2}=L|K)\pi_{K}.$$

But $P(K_{1}=L|K)=P(K_{2}=L|K)$, hence

$$\begin{array}{ll} P({\hat{K}}_{1}={\hat{K}}_{2}) & =\underset{K\in 𝒦}{\sum }\underset{L\in 𝒦}{\sum }P{\left(\hat{K}=L|K\right)}^{2}\pi_{K}\;\\ & =\underset{K\in 𝒦}{\sum }\underset{L\in 𝒦}{\sum }{\left(\underset{R\in {ℛ}_{L}}{\sum }\frac{P(R|K)}{\left|{𝒦}_{R}\right|}\right)}^{2}\pi_{K}.\; \end{array}$$

It is interesting to notice that an estimate of this probability can be obtained on the basis of the estimated $\beta_{q}$, $\eta_{q}$ and $\pi_{K}$ parameters, without the need to produce the concrete test‐retest situation. Moreover, it might well be possible that ${\hat{K}}_{1}={\hat{K}}_{2}$, but ${\hat{K}}_{1}\neq K$, that is neither the first, nor the second assessment coincide with the true state, although they are identical. It would thus be interesting to study the relationship between the single assessment accuracy rate $P(\hat{K}=K)$ and the test‐retest accuracy rate $P({\hat{K}}_{1}={\hat{K}}_{2})$. However, this investigation is not pursued in the present article.

## AUTHOR CONTRIBUTIONS

**Debora de Chiusole:** conceptualization; methodology; formal analysis; writing – original draft; writing – review and editing; project administration. **Andrea Spoto:** conceptualization; methodology; writing – original draft; writing – review and editing; supervision. **Umberto Granziol:** writing – original draft; writing – review and editing; conceptualization; methodology. **Luca Stefanutti:** conceptualization; methodology; supervision; writing – original draft; writing – review and editing.

## CONFLICT OF INTEREST STATEMENT

None of the authors have a conflict of interest to declare.

## DISCLOSURE OF ARTIFICIAL INTELLIGENCE‐GENERATED CONTENT (AIGC) TOOLS

No Artificial Intelligence‐Generated Content (AIGC) tools were used in the preparation of this work.

## Supporting information


Appendix S1.

## Data Availability

The data that support the findings of this study are openly available in Open Science Framework at https://osf.io/nr2ug/?view_only=ea8b0914f6a74ed28a84e60547c72b2d.

## Footnotes

^1^
Notation |X| denotes the cardinality of set X, that is the number of elements contained in it.
