# Supplementary material for: Reliability measures in knowledge structure theory
Source: Br J Math Stat Psychol. 2025 Nov 1;79(3):457–78. doi: 10.1111/bmsp.70013 (PMC13679758; doi:10.1111/bmsp.70013)
Supplement: Supplementary file 1 [file BMSP-79-457-s001.pdf]

Web Supplementary Material of the article "Reliability Measures in Knowledge Structure Theory"

Supplementary Material of Study 1

Figures 1, 2, 3, and 4 show the results about the accuracy rate (solid lines) and the expected discrepancy $E(\mathbf{D})$ (dashed lines) across varying levels of noise (x-axis), when the knowledge state probability distribution is random non-uniform, random right-asymmetric, random left-asymmetric, and random symmetric, respectively. Each panel corresponds to a different cardinality of the knowledge structure $|K_{ij}|$, ranging from 2 to 200. The results are shown for domains with three different item numbers: $|Q_i| = 10$ (blue lines), $|Q_i| = 15$ (orange lines), and $|Q_i| = 20$ (yellow lines).

Figures 5, 6, 7, and 8 show the performance of the ρ accuracy rate (panels on the left) and the expected discrepancy $E(\mathbf{D})$ (panels on the right), when the knowledge state probability distribution is random non-uniform, random right-asymmetric, random left-asymmetric, and random symmetric respectively. The results are shown for different cardinalities of the structure (represented by different line colors in each panel). Each row of the figure displays the results obtained with a different number of items: $|Q_i| = 10$ (panels to the top), $|Q_i| = 15$ (panels in the middle), and $|Q_i| = 20$ (panels to the bottom).

Figures 9, 10, 11, and 12 compares KS-reliability (y-axis) with accuracy rate (x-axis)

across different knowledge structure cardinalities (panels in the figure) and numbers of items (the three colored lines).

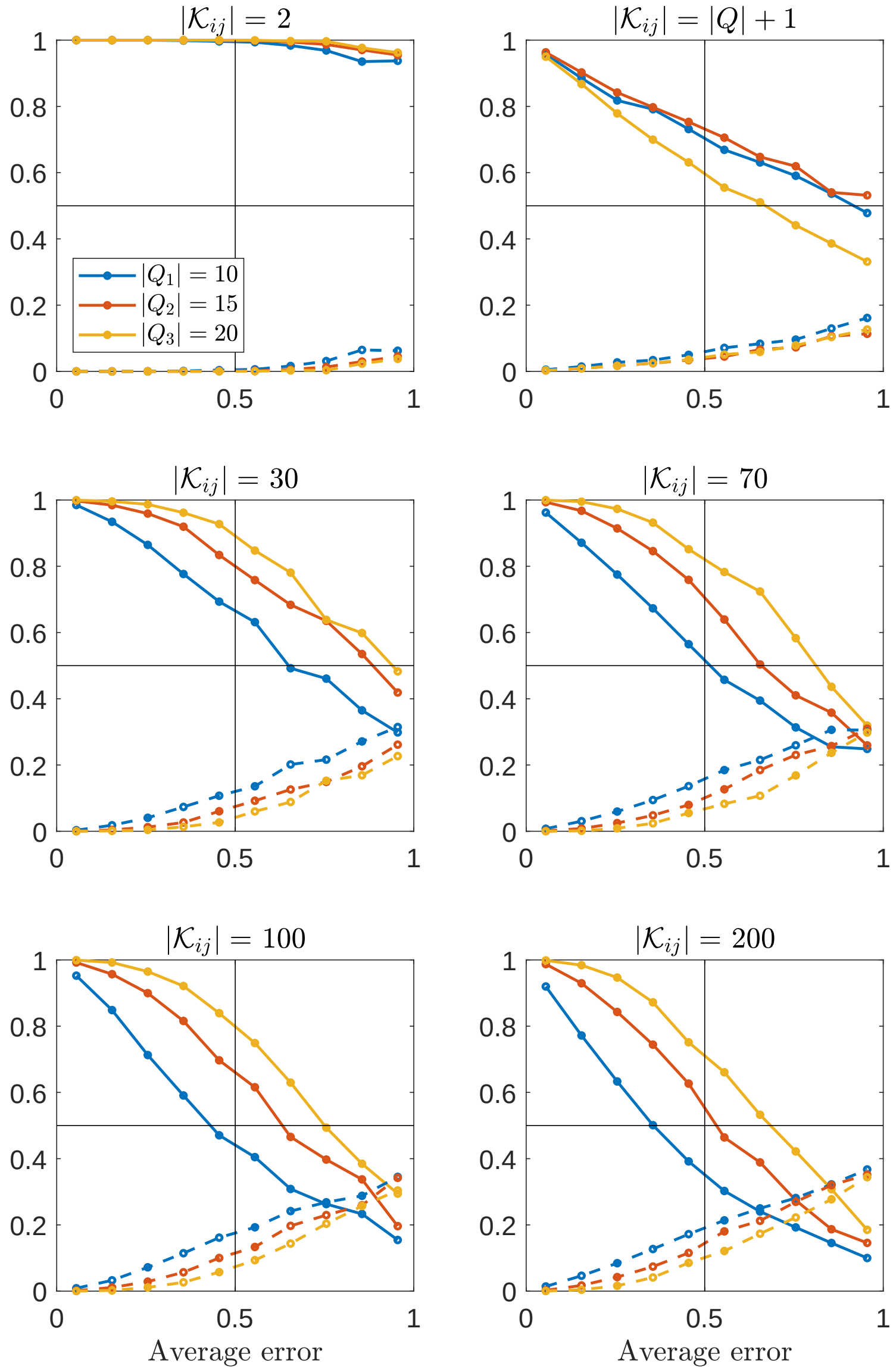

Figure 1: Random non-uniform: Comparison of accuracy rate ρ (solid lines) and expected discrepancy $E(\mathbf{D})$ (dashed lines) across different cardinalities of the knowledge structure (panels of the figures) and noise levels (x-axis).

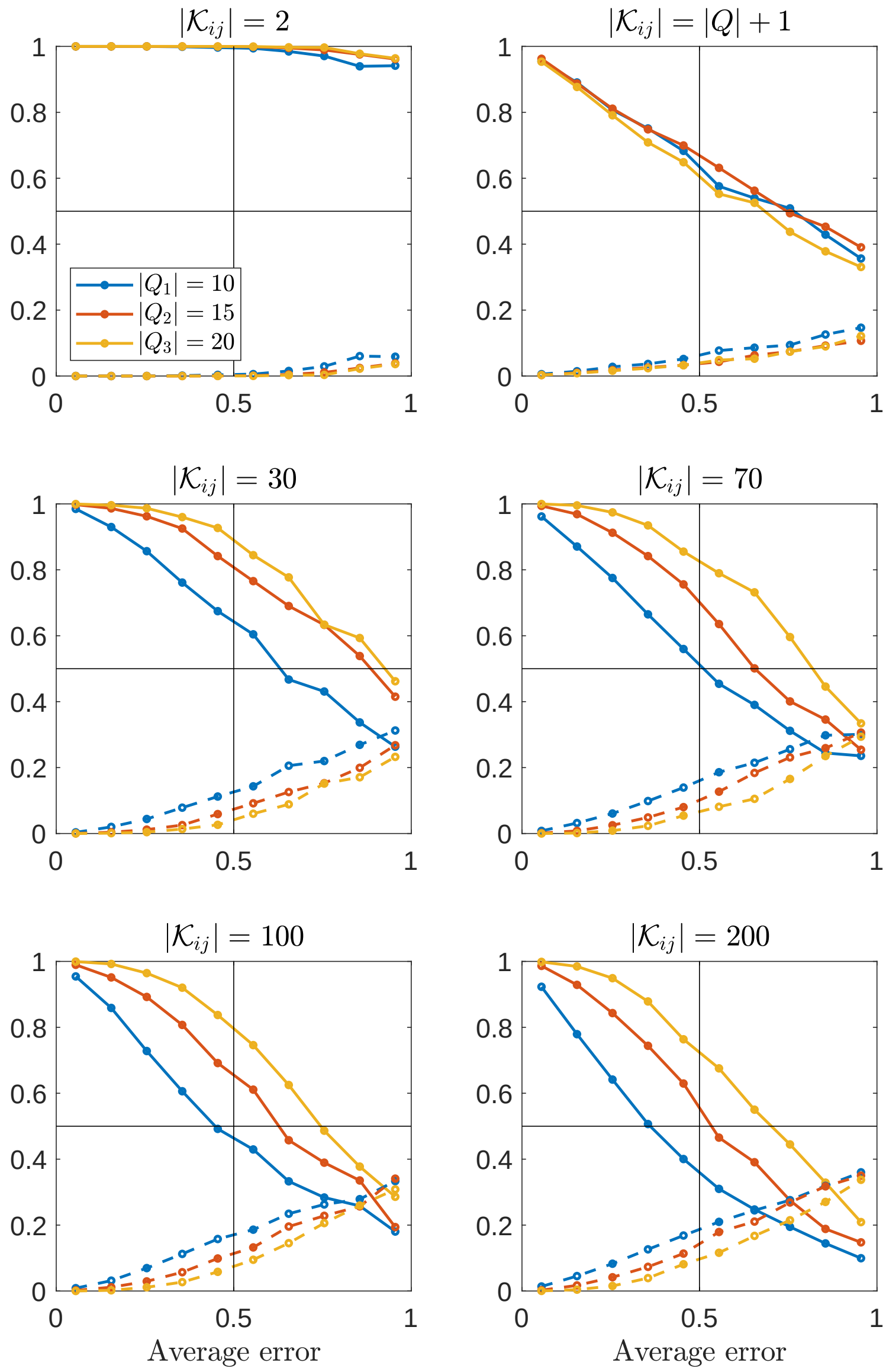

Figure 2: Random left-asymmetric: Comparison of accuracy rate ρ (solid lines) and expected discrepancy $E(\mathbf{D})$ (dashed lines) across different cardinalities of the knowledge structure (panels of the figures) and noise levels (x-axis).

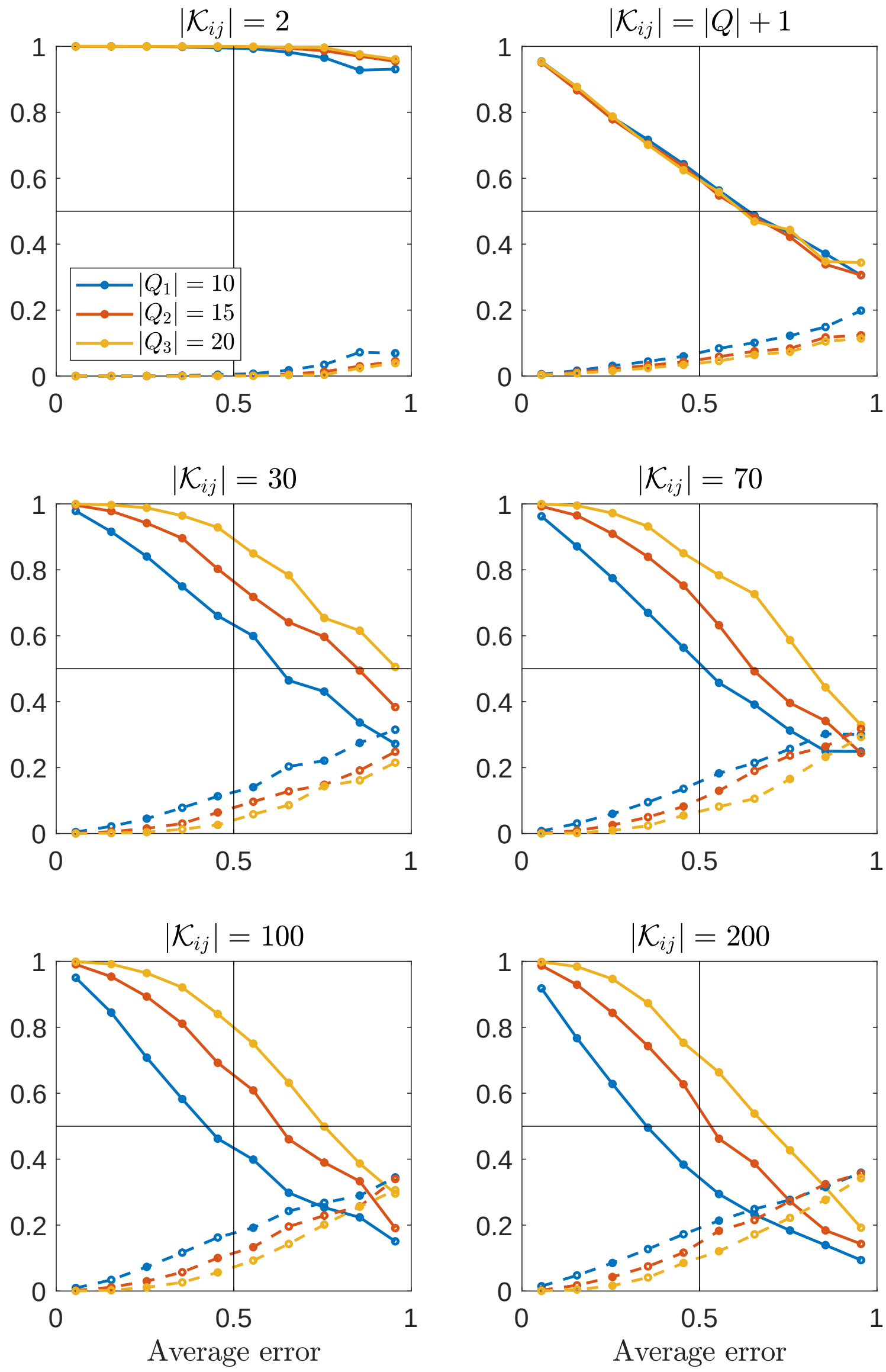

Figure 3: Random right-asymmetric: Comparison of accuracy rate ρ (solid lines) and expected discrepancy $E(\mathbf{D})$ (dashed lines) across different cardinalities of the knowledge structure (panels of the figures) and noise levels (x-axis).

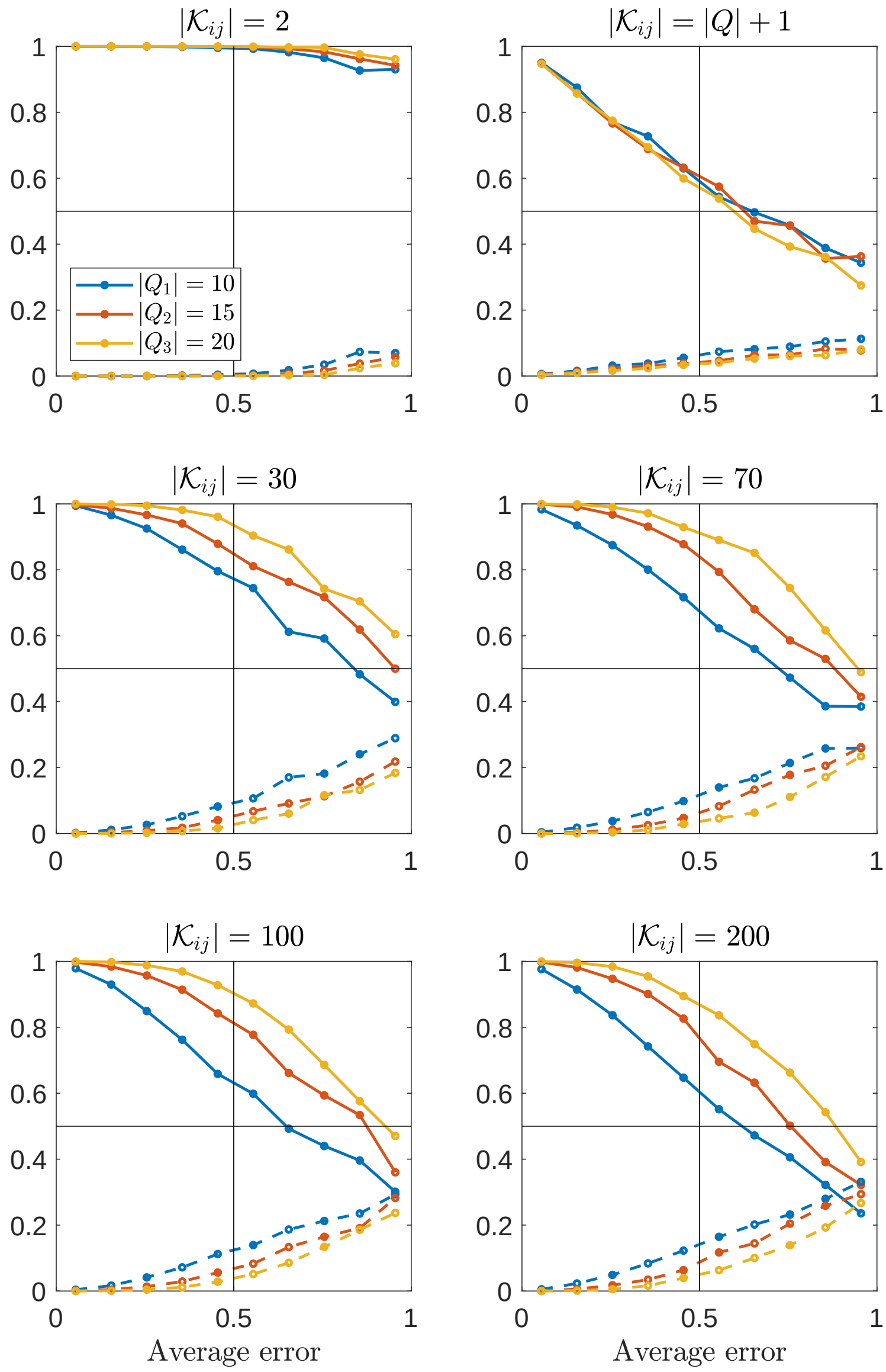

Figure 4: Random symmetric: Comparison of accuracy rate ρ (solid lines) and expected discrepancy $E(\mathbf{D})$ (dashed lines) across different cardinalities of the knowledge structure (panels of the figures) and noise levels (x-axis).

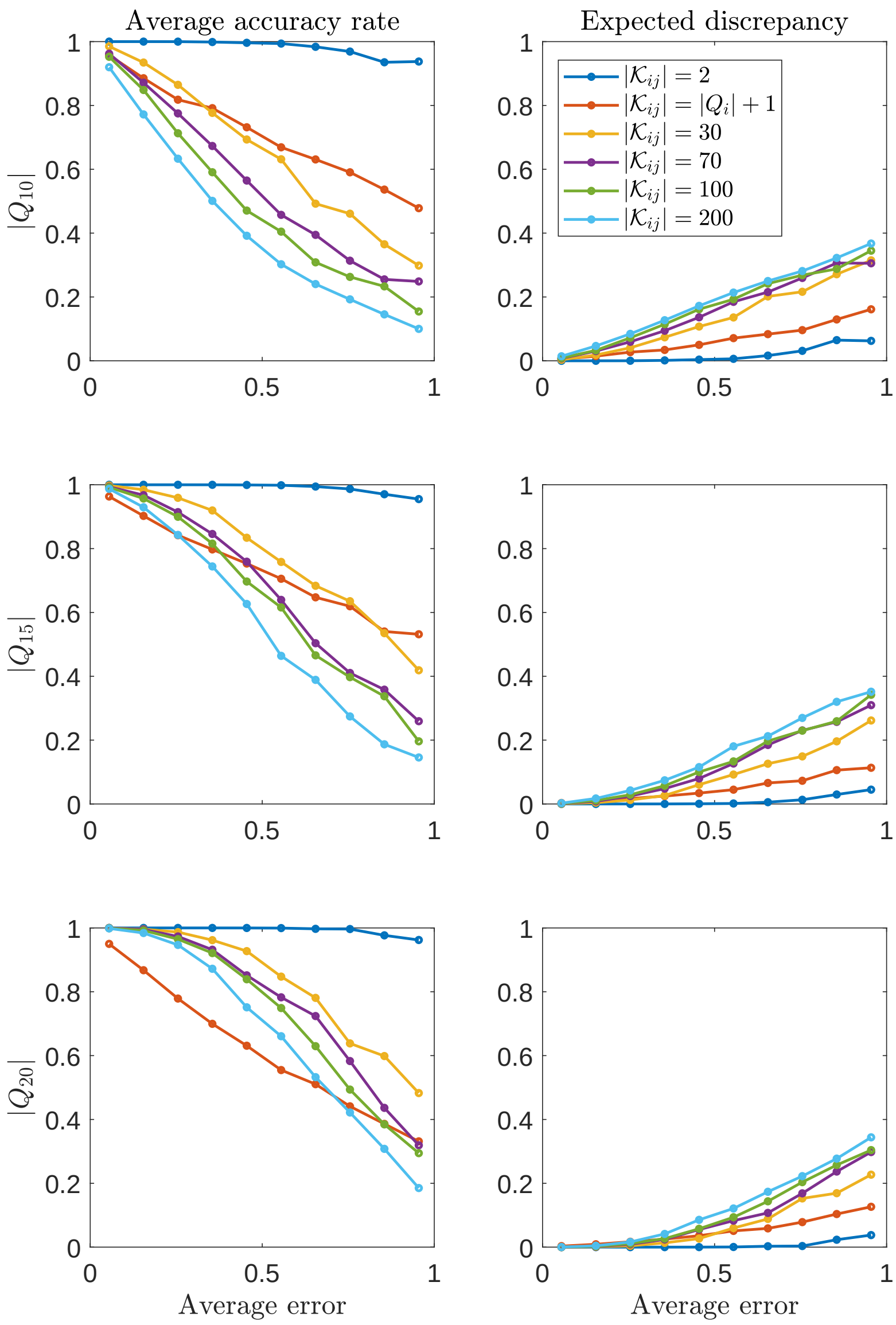

Figure 5: Random non-uniform: Comparison of accuracy rate (on the left) and expected discrepancy (on the right) across different cardinalities of the knowledge structure (colored lined in each panel) and noise levels (x-axis). Panels display results for varying numbers of items in the domain: $|Q| = 10$ (top panels), 15 (middle panels), 20 (bottom panels).

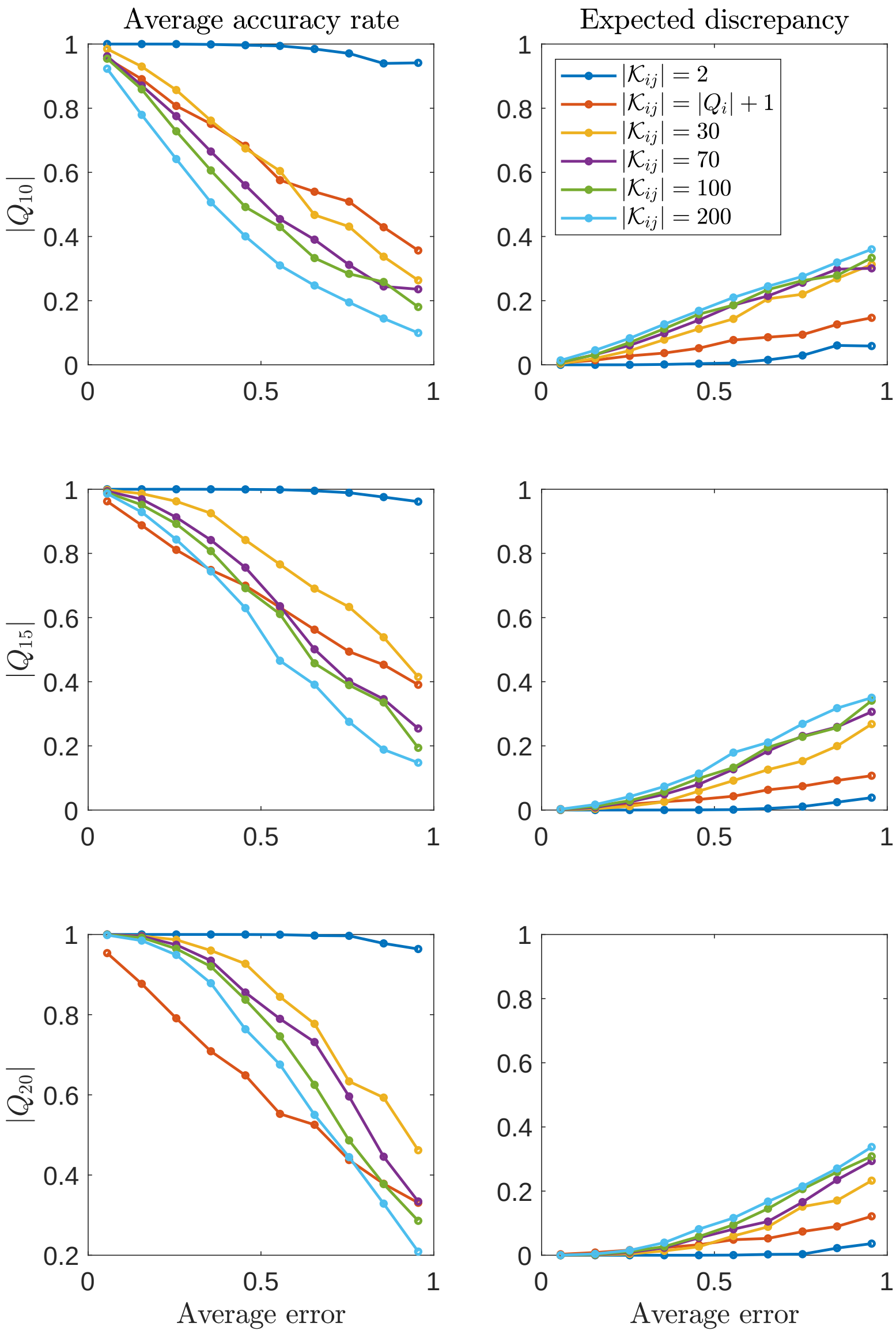

Figure 6: Random right-asymmetric: Comparison of accuracy rate (on the left) and expected discrepancy (on the right) across different cardinalities of the knowledge structure (colored lined in each panel) and noise levels (x-axis). Panels display results for varying numbers of items in the domain: $|Q| = 10$ (top panels), 15 (middle panels), 20 (bottom panels).

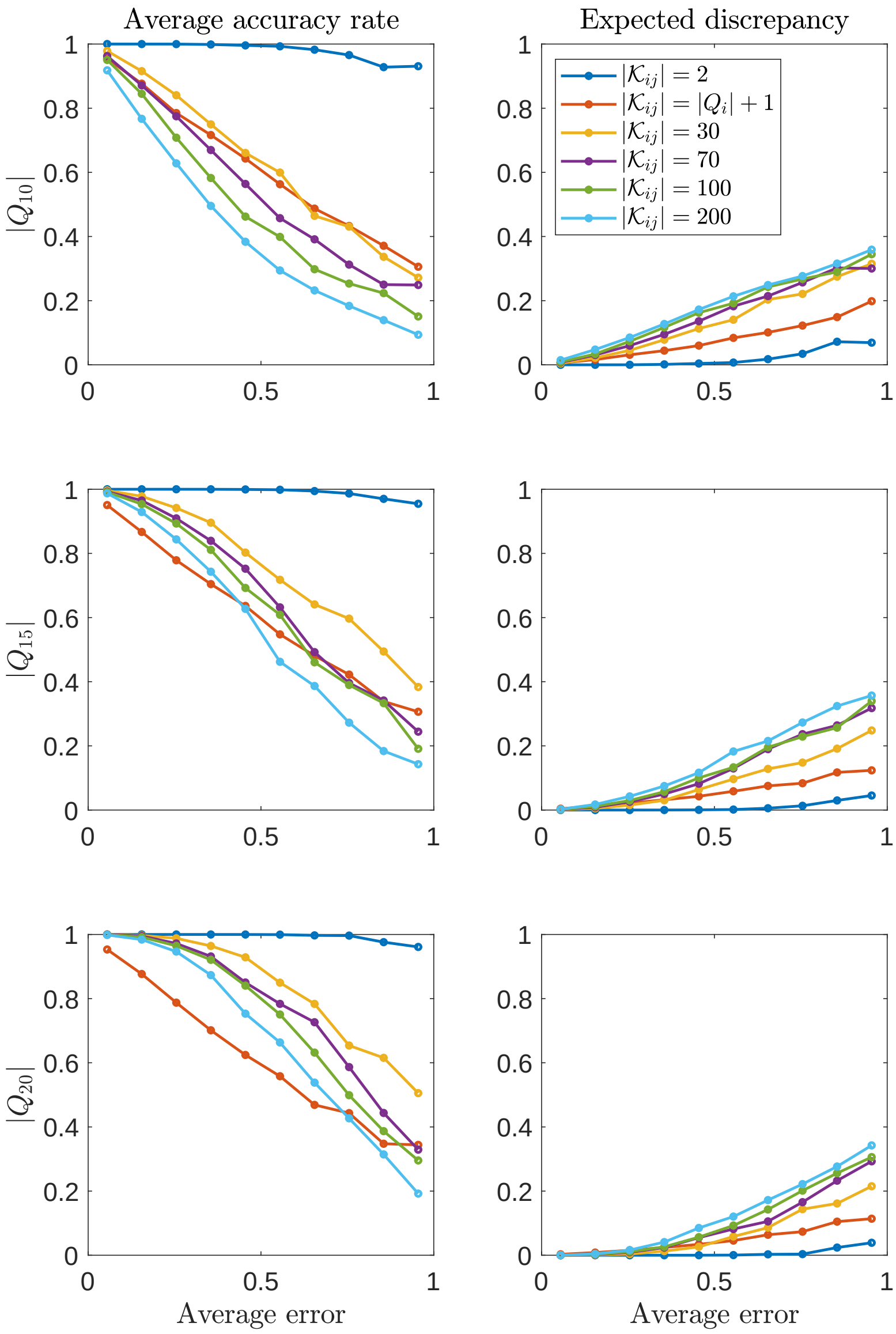

Figure 7: Random right-asymmetric: Comparison of accuracy rate (on the left) and expected discrepancy (on the right) across different cardinalities of the knowledge structure (colored lined in each panel) and noise levels (x-axis). Panels display results for varying numbers of items in the domain: $|Q| = 10$ (top panels), 15 (middle panels), 20 (bottom panels).

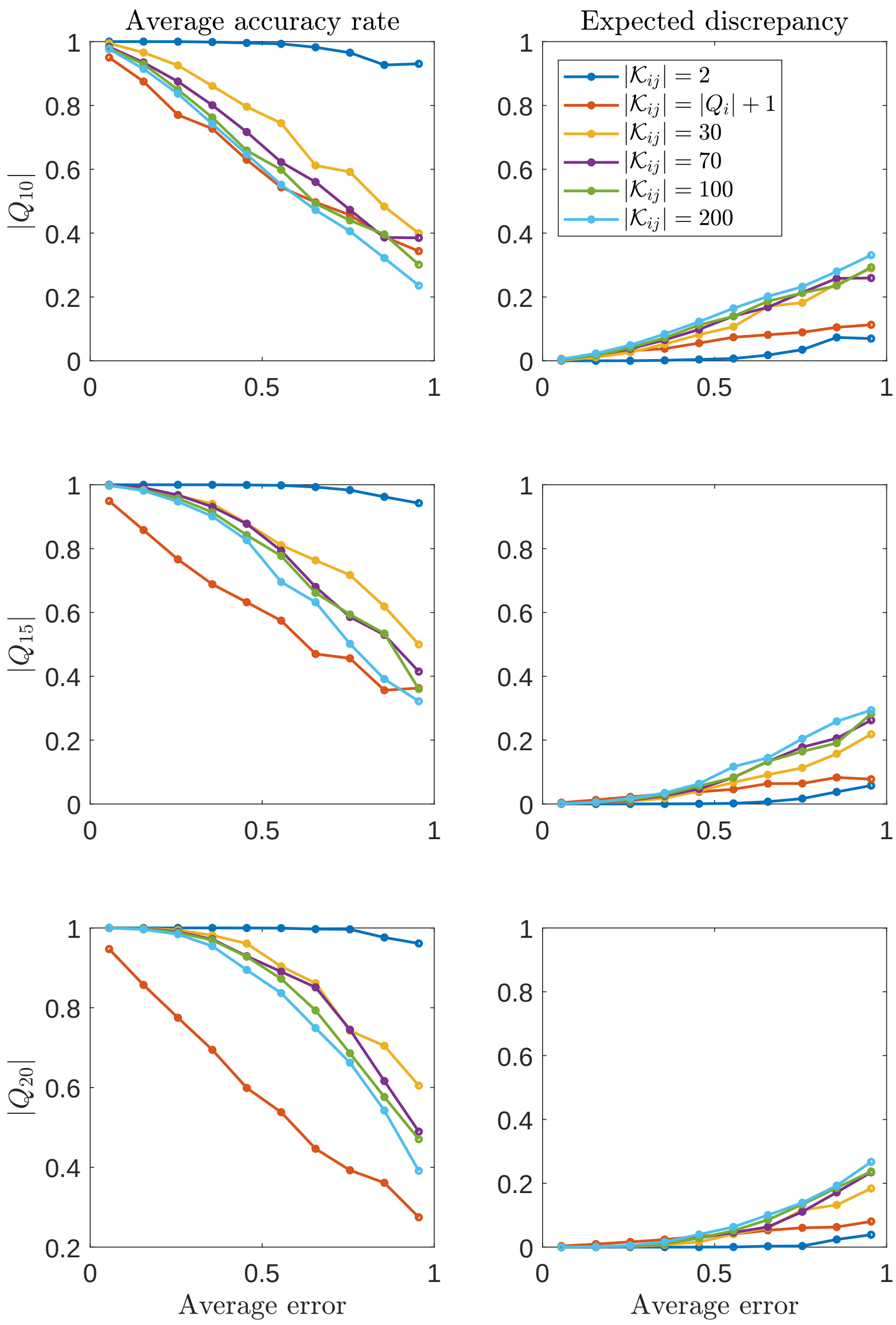

Figure 8: Random symmetric: Comparison of accuracy rate (on the left) and expected discrepancy (on the right) across different cardinalities of the knowledge structure (colored lined in each panel) and noise levels (x-axis). Panels display results for varying numbers of items in the domain: $|Q| = 10$ (top panels), 15 (middle panels), 20 (bottom panels).

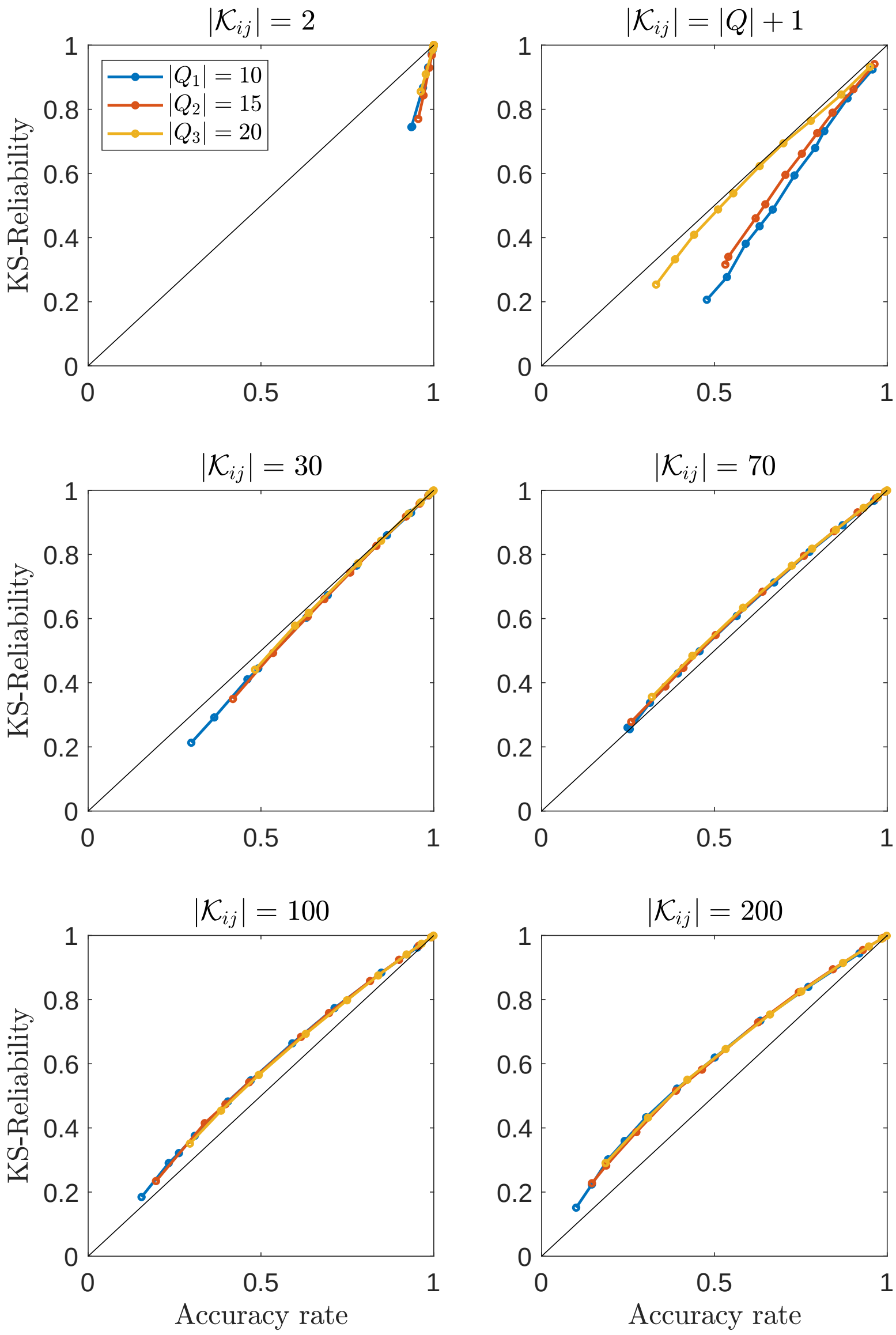

Figure 9: Random right-asymmetric: Comparison of accuracy rate (x-axis) and KS-Reliability (y-axis) across different cardinalities of the knowledge structure (panels of the figure) and number of items (the three colored lines).

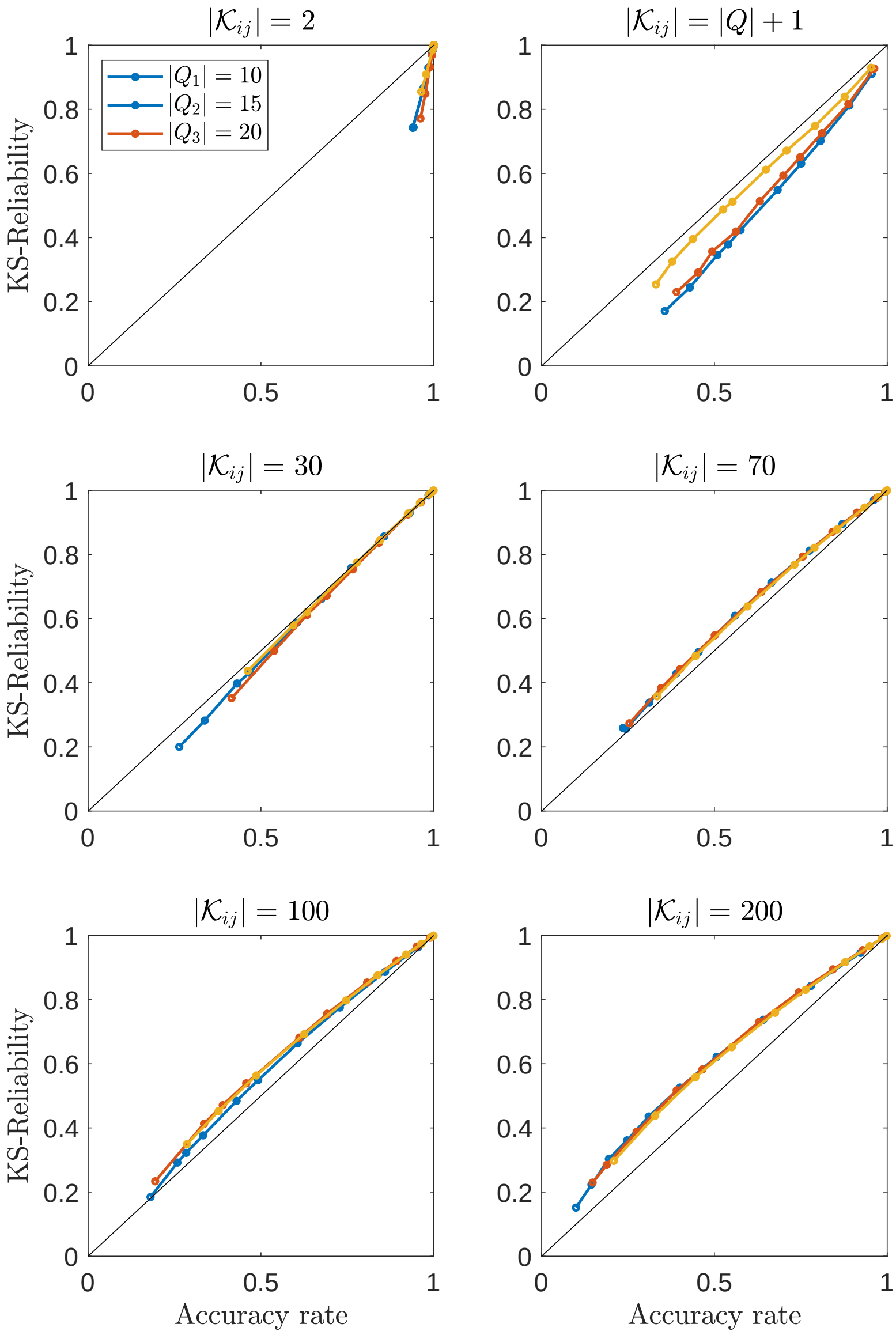

Figure 10: Random right-asymmetric: Comparison of accuracy rate (x-axis) and KS-Reliability (y-axis) across different cardinalities of the knowledge structure (panels of the figure) and number of items (the three colored lines).

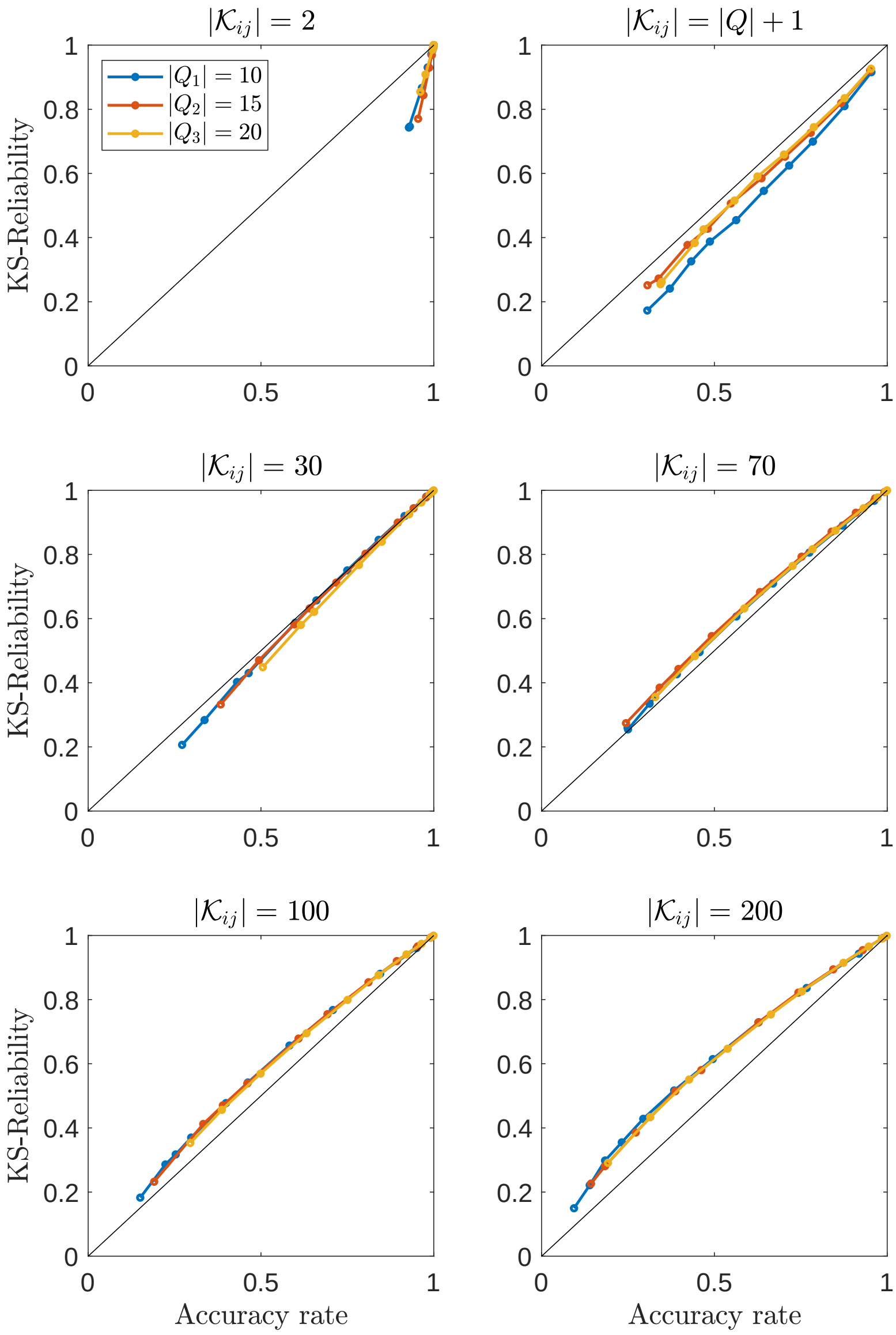

Figure 11: Random right-asymmetric: Comparison of accuracy rate (x-axis) and KS-Reliability (y-axis) across different cardinalities of the knowledge structure (panels of the figure) and number of items (the three colored lines).

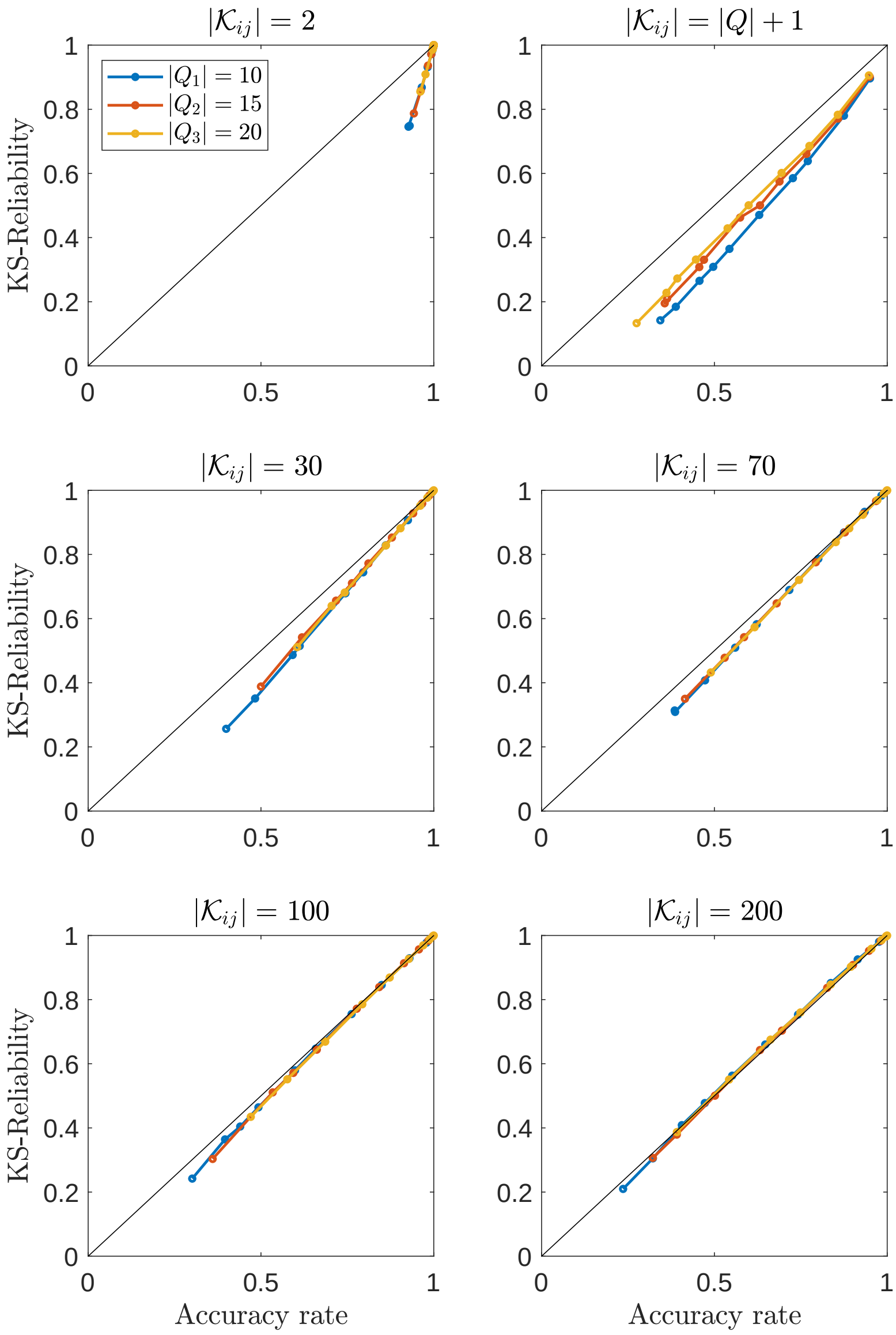

Figure 12: Random symmetric: Comparison of accuracy rate (x-axis) and KS-Reliability (y-axis) across different cardinalities of the knowledge structure (panels of the figure) and number of items (the three colored lines).
